# Identification of potentially actionable genetic variants in epithelial ovarian cancer: a retrospective cohort study

**DOI:** 10.1038/s41698-024-00565-2

**Published:** 2024-03-22

**Authors:** Charlotte Fieuws, Joni Van der Meulen, Kristiaan Proesmans, Emiel A. De Jaeghere, Siebe Loontiens, Jo Van Dorpe, Philippe Tummers, Hannelore Denys, Koen Van de Vijver, Kathleen B. M. Claes

**Affiliations:** 1 Department of Biomolecular Medicine, Ghent University, Ghent, Belgium; 2 Center for Medical Genetics Ghent, Ghent University Hospital, Ghent, Belgium; 3 Cancer Research Institute Ghent, Ghent, Belgium; 4 Department of Bio-Analysis, Ghent University, Ghent, Belgium; 5 Department of Medical Oncology, Ghent University Hospital, Ghent, Belgium; 6 Department of Pathology, Ghent University Hospital, Ghent, Belgium

**Keywords:** Ovarian cancer, Targeted therapies, Cancer genomics, Tumour heterogeneity

## Abstract

Ovarian cancer is the most lethal gynecologic malignancy, mainly due to late-stage diagnosis, frequent recurrences, and eventually therapy resistance. To identify potentially actionable genetic variants, sequencing data of 351 Belgian ovarian cancer patients were retrospectively captured from electronic health records. The cohort included 286 (81%) patients with high-grade serous ovarian cancer, 17 (5%) with low-grade serous ovarian cancer, and 48 (14%) with other histotypes. Firstly, an overview of the prevalence and spectrum of the *BRCA1/2* variants highlighted germline variants in 4% (11/250) and somatic variants in 11% (37/348) of patients. Secondly, application of a multi-gene panel in 168 tumors revealed a total of 214 variants in 28 genes beyond *BRCA1/2* with a median of 1 (IQR, 1–2) genetic variant per patient. The ten most often altered genes were (in descending order): *TP53*, *BRCA1*, *PIK3CA*, *BRCA2*, *KRAS*, *ERBB2* (*HER2*), *TERT promotor*, *RB1*, *PIK3R1* and *PTEN*. Of note, the genetic landscape vastly differed between the studied histotypes. Finally, using ESCAT the clinical evidence of utility for every genetic variant was scored. Only *BRCA1/2* pathogenic variants were classified as tier-I. Nearly all patients (151/168; 90%) had an ESCAT tier-II variant, most frequently in *TP53* (74%), *PIK3CA* (9%) and *KRAS* (7%). In conclusion, our findings imply that although only a small proportion of genetic variants currently have direct impact on ovarian cancer treatment decisions, other variants could help to identify novel (personalized) treatment options to address the poor prognosis of ovarian cancer, particularly in rare histotypes.

## Introduction

Epithelial ovarian cancer (OC) is an umbrella term for distinct malignancies affecting the ovaries, each characterized by its own histological and molecular profile. OC is the most lethal gynecological malignancy and the eighth most prevalent female cancer worldwide, with an annual abysmal mortality rate of two million fatalities worldwide^1^. The existence of late symptom onset and multiple overlapping symptoms with other gastrointestinal, genitourinary, and gynecological diseases often leads to late-stage diagnosis with already extensive extra-ovarian metastasis present, whereby the 5 year survival plummets to <30%^2–5^. Despite their inherent differences, most OCs are generally treated in the same manner: the cornerstones are cytoreductive surgery and platinum-based chemotherapy, with or without Poly (ADP-Ribose) Polymerase (PARP) inhibitors (PARPis) and/or bevacizumab^6^. Unfortunately, up to 70% of patients will experience recurrence^7^. Thus, it is well established that a “one-size-fits-all” treatment approach should no longer be applied. Tailored treatment options based on individualized molecular tumor data are warranted to enable practitioners to more effectively treat specific OC subtypes. For instance, EMA and FDA have approved PARPis targeting germline or somatic *BRCA1/2* mutated advanced OCs. Given their efficacy, mutational screening of *BRCA1/2* genes in OC has become of great importance^8–11^. Besides PARPis, targeted therapies are increasingly available for other gene alterations, including KRAS, BRAF and TRK inhibitors^12^. Therefore, implementation of companion diagnostic tests, which go beyond somatic and germline *BRCA1/2* testing is highly warranted.

To help clinicians with interpreting genomic reports, the European Society for Medical Oncology (ESMO), Translational Research and Precision Medicine Working Group published a systemic framework in 2018 to rank molecular targets based on available evidence supporting their value as clinical targets^13^. Its main goal was to implement a harmonized vocabulary and to facilitate communication between academia, pharmaceutical industry, healthcare professionals and patients. The ESMO scale for Clinical Actionability of Molecular Targets (ESCAT) was previously applied across multiple cancer types, but OC has never been the focus of such studies and histopathologic subtypes were never distinguished^14–17^.

In the present retrospective cohort study, we aim to characterize (potentially targetable) genetic variants in patients with OC across various histotypes, using the ESCAT framework.

## Results

### Patients and samples

**Fig. 1:**
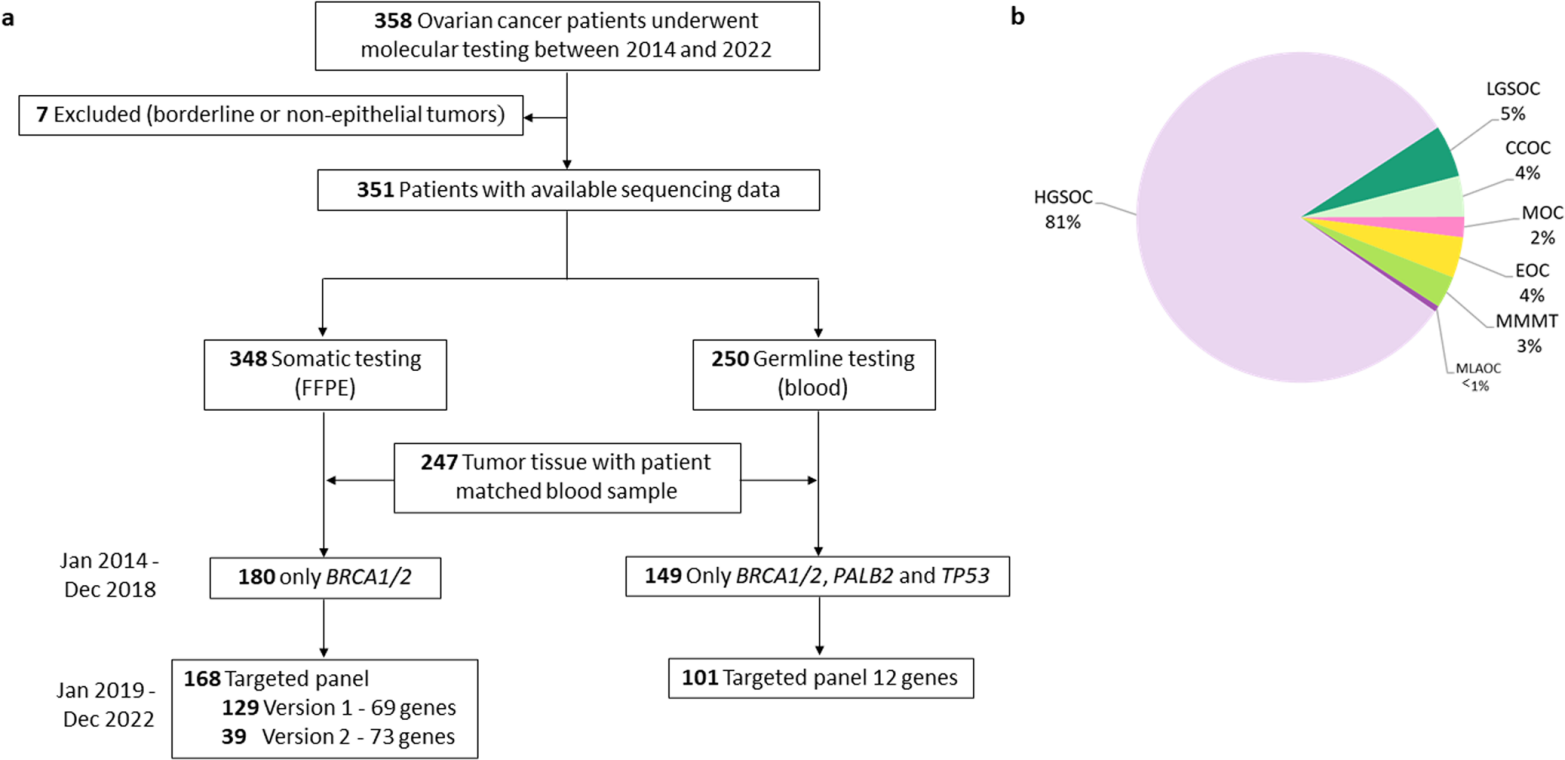
Overview of the study cohort. **a** Molecular workup of blood and tumor samples. **b** Histotype distribution.

HE slides from 358 OC patients (median age at molecular analysis was 68 year, range 29–90 years) who received surgery between 2014 and 2022 were reviewed by an expert pathologist; seven borderline or non-epithelial tumors were excluded for further analysis (*n* = 7). From the final cohort of 351 patients, sequencing data were available from 348 tumor samples and 250 blood samples. Patient matched tumor and blood samples were available for 247 patients (Fig. 1a). The most common histotypes were high-grade serous ovarian cancer (HGSOC) (*n* = 286) and low-grade serous ovarian cancer (LGSOC) (*n* = 17), with other (rare) histotypes accounting for 48 patients: 15 clear cell carcinoma of the ovaries (CCOC), 14 endometrioid ovarian cancers (EOC), 11 mixed malignant mullerian tumors (MMMT), 6 mucinous ovarian cancers (MOC) and 2 mesonephric-like adenocarcinomas of the ovary (MLAOC) (Fig. 1b).

### Genetic variants in *BRCA1/2*

Among the 351 OC patients analyzed, 49 (14%) patients harbored *BRCA1* (9%) and/or *BRCA2* (5%) variants. In one HGSOC patient, both a somatic *BRCA1* and a germline *BRCA2* variant were detected. Eleven variants were of germline origin (six in *BRCA1* and five in *BRCA2*). *BRCA1/2* variants were most prevalent in the HGSOC (16%) subgroup; notably, all germline variants were found in this histotype. The remaining somatic *BRCA1/2* variants were found in CCOC (13%), MMMT (9%) and EOC (7%) histotypes. No *BRCA1/2* variants were found in the LGSOC, MOC, and MLAOC histotypes (Table 1).

**Table 1:** BRCA1/2 variant distribution according to histotypes (*n* = 351)

	*BRCA1*		*BRCA2*		Total *BRCA1* + *2*	
	somatic	germline	somatic	germline	somatic	germline
HGSOC	24/283	6/216	11/282	5/216	35/283	11/216
LGSOC	0/17	0/12	0/17	0/12	0/17	0/12
CCOC	1/15	0/4	1/15	0/4	2/15	0/4
EOC	1/14	0/7	0/14	0/7	1/14	0/7
MMMT	1/11	0/7	0/11	0/7	1/11	0/7
MOC	0/6	0/4	0/6	0/4	0/6	0/4
MLAOC	0/2	NA	0/2	NA	0/2	NA
total	**27/348** **7.8%**	**6/250** **2.4%**	**12/347** **3.5%**	**5/250** **2.0%**	**39/348** **11.26%**	**11/250** **4.4%**

**Fig. 2:**
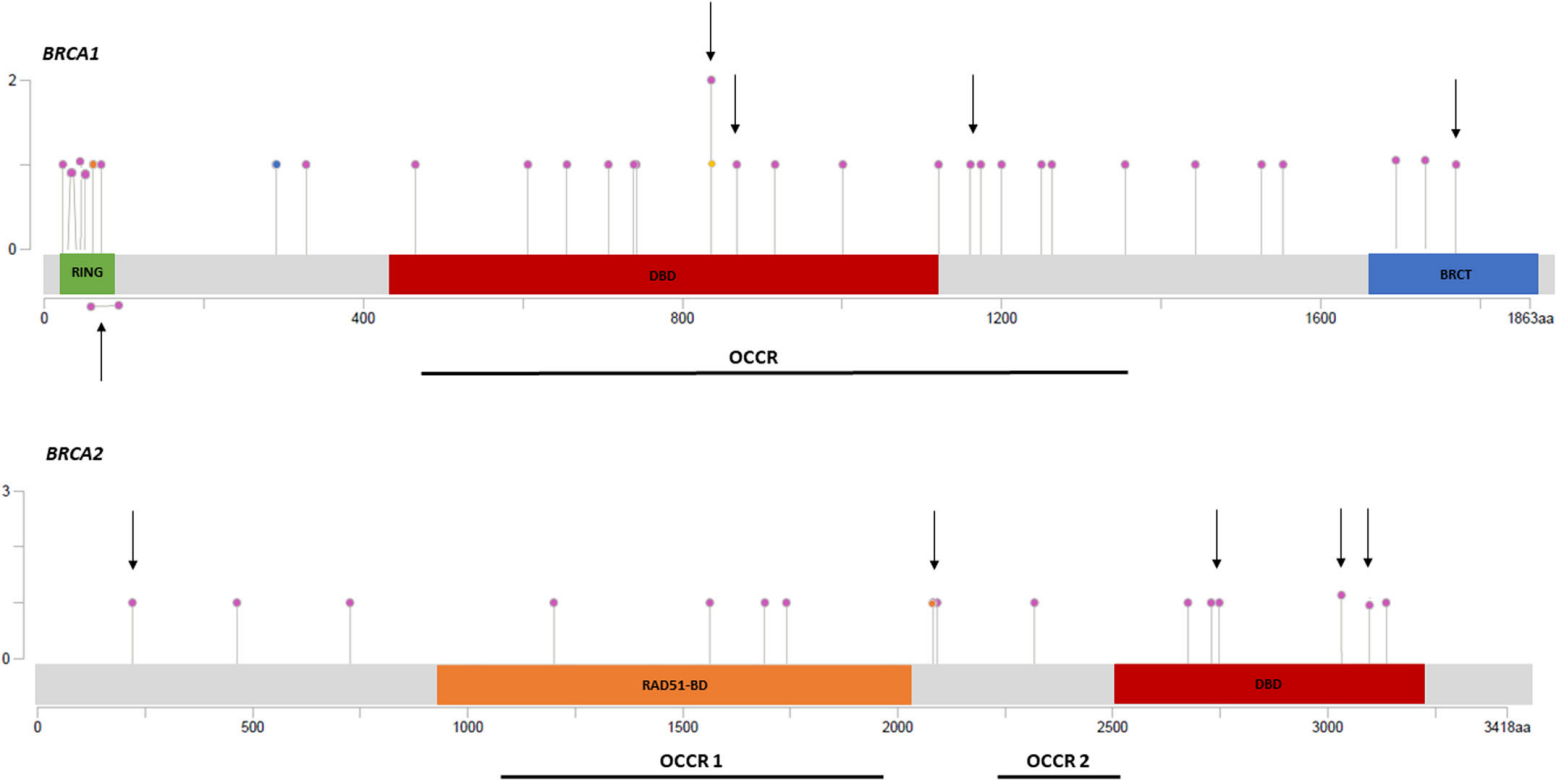
Lollipop plots of variant distribution in BRCA1 and BRCA2. The diagrams linearly represent BRCA1/2 protein domains (*x*-axis). BRCA1 domains: green, C3HC4-type RING finger; red, DBD (DNA-binding domain of BRCA1); blue, BRCT (BRCA1 C-terminal) domain. BRCA2 domains: orange, RAD51-BD (RAD51-binding domain); red, DBD (DNA-binding domain). Each variant is represented by a single lollipop; the stick heights indicate the mutation frequency (*y*-axis), and dots are color-coded according to histotype: pink dots, HGSOC; green dots, LGSOC; orange dots, CCOC; blue dots, MMMT; yellow dots, EOC. Germline variants are indicated by an arrow. The deletion of exons 5 & 6 in BRCA1 is marked by a horizontal lollipop under the plot. OCCR (ovarian cancer cluster regions) are indicated by a thick black line under the plot. The graphs are adapted from MutationMapper tool, cBioportal (http://www.cbioportal.org/mutation_mapper).

To evaluate if the variants cluster in specific regions, all (likely) pathogenic variants are depicted in a lollipop plot, illustrating that the variants were clearly spread over the entire coding sequence of *BRCA1* and *BRCA2* genes, without particular hotspot regions (Fig. 2). Less than half of the variants were found in the ovarian cluster regions (OCCR): sixteen in the *BRCA1* OCCR (13/27 somatic, 3/6 germline), four in *BRCA2* OCCR1 and one in *BRCA2* OCCR2 (all somatic). Twenty-two (6% of patients) and eleven (3% of patients) variants occurred in a functional domain of *BRCA1* and *BRCA2*, respectively. In *BRCA1*, this concerns seven variants in the Really Interesting New Gene (RING) domain, twelve in the DNA Binding Domain (DBD) and three in the BRCA1 C-Terminal (BRCT) domain. In *BRCA2*, four variants clustered in the RAD51-Binding Domain (RAD51-BD) and seven in the DBD. A detailed overview of all germline and somatic *BRCA1/2* variants identified in the different histotypes is provided in Supplementary Table 1.

#### Genetic variants beyond *BRCA1/2*

A multi-gene panel was introduced in 2019 for solid cancers and revealed a wide range of genetic variants in 168 OCs. Beyond *BRCA1/2*, 214 variants across 27 genes were detected in 134 patients (80%) with a median of 1 variant (interquartile range (IQR), 1–2) per patient. The majority of these variants were substitutions (164/214, 77%), small deletions (33/214, 15%), or duplications (9/214, 4%). In a limited number of samples, coverage analysis revealed gene amplifications: *PIK3CA* (2%), *ERBB2* (2%), *FGFR2* (1%), *FGFR3* (1%), *MET* (1%), *CCND1* (1%). Sixty-six tumors harbored more than one (likely) pathogenic variant in the same gene or multiple genes. Table 2 displays an overview of the most frequently altered genes per histotype. A complete list of all variants found in our cohort is provided in Supplementary Table 2.

**Table 2:** Overview of the frequency of altered genes according to histotypes

*n* (%)	Total 168 (100)	HGSOC 125 (74.4)	CCOC 15 (8.9)	LGSOC 11 (6.5)	EOC 7 (4.2)	MMMT 6 (3.6)	MOC 2 (1.2)	MLAOC 2 (1.2)
*TP53*	127 (75.6)	117 (93.6)	2 (13.4)	0	3 (42.9)	4 (66.7%)	1 (50.0)	0
*PIK3CA*	15 (8.9)	6 (4.8)	6 (40.0)	0	2 (28.6)	0	1(50.0)	0
*KRAS*	11 (6.5)	3 (2.4)	2 (13.4)	1 (9.1)	1 (14.3)	0	2 (100)	2 (100)
*ERBB2*	5 (3.0)	2 (1.6)	0	3 (27.3)	0	0	0	0
*TERT promotor*	4 (2.4)	0	4 (26.7)	0	0	0	0	0
*RB1*	4 (2.4)	4 (3.2)	0	0	0	0	0	0
*PIK3R1*	4 (2.4)	3 (2.4)	1 (6.7)	0	0	0	0	0
*PTEN*	4 (2.4)	1 (<1.0)	1 (6.7)	0	2 (28.6)	0	0	0
*ATM*	3 (1.8)	0	2 (13.4)	0	1 (14.3)	0	0	0
*NRAS*	3 (1.8)	0	0	3 (27.3)	0	0	0	0
*FGFR2*	2 (1.2)	2 (1.6)	0	0	0	0	0	0
*MET*	2 (1.2)	2 (1.6)	0	0	0	0	0	0
*CCND1*	2 (1.2)	0	1 (6.7)	0	1 (14.3)	0	0	0
*CDKN2A*	2 (1.2)	0	1 (6.7)	1 (9.1)	0	0	0	0
*AKT1*	2 (1.2)	1 (<1.0)	0	0	1 (14.3)	0	0	0
*CTNNB1*	2 (1.2)	0	0	0	2 (28.6)	0	0	0
*RNF43*	2 (1.2)	2 (1.6)	0	0	0	0	0	0
*FGFR3*	1 (<1.0)	1 (<1.0)	0	0	0	0	0	0
*BRAF*	1 (<1.0)	0	0	1 (9.1)	0	0	0	0
*FGFR1*	1 (<1.0)	1 (<1.0)	0	0	0	0	0	0
*SMARCA4*	1 (<1.0)	1 (<1.0)	0	0	0	0	0	0
*SMARCB1*	1 (<1.0)	0	1 (6.7)	0	0	0	0	0
*GNAS*	1 (<1.0)	1 (<1.0)	0	0	0	0	0	0
*POLE*	1 (<1.0)	0	1 (6.7)	0	0	0	0	0
*SPOP*	1 (<1.0)	1 (<1.0)	0	0	0	0	0	0
*BAP1*	1 (<1.0)	0	0	1 (9.1)	0	0	0	0
*VHL*	1 (<1.0)	1 (<1.0)	0	0	0	0	0	0

The table shows the number of tumors with at least one variant in the corresponding gene.

Summarized, in HGSOC, the most frequently altered gene was *TP53* in 94% of patients. Other affected genes include *PIK3CA* (4.8%), *RB1* (3.2%), *KRAS* (2.4%), and *PIK3R1* (2.4%). Sporadically altered genes (only 1 or 2 tumors) in this subtype are *RNF43, FGFR2, ERBB2, MET, AKT1, PTEN, GNAS, FGFR1, FGFR3, SMARCA4, VHL* and *SPOP*. In fifteen CCOC tumors, variants were identified in eleven genes with *PIK3CA* being mutated in six (40%) and the *TERT* promotor in four (27%) tumors, respectively. Other genes (*TP53, ATM, PIK3R1, PTEN, KRAS, CCND1, CDKN2A, SMARCB1 and POLE)* were only sporadically affected in CCOC. Ten out of eleven LGSOC tumors had variants identified in *NRAS* (27%), *ERBB2* (27%) and sporadically in *BAP1*, *KRAS*, *CDKN2A* and *BRAF*. Six out of seven EOC tumors harbored variants in *TP53*, *AKT1*, *PIK3CA*, *PTEN*, *KRAS*, *CCND1* and *CTNNB1*. In three EOC tumors multiple variants were detected (one tumor with six variants, another had 3 variants and a third had two variants). Out of six carcinosarcomas, three had variants in *TP53*. Both mucinous and mesonephric-like adenocarcinomas tumors harbored the same *KRAS* p.(Gly12Asp) variant. Additionally, one mucinous tumor harbored a *TP53* variant and another had a *PIK3CA* variant.

### Affected signaling pathways

**Fig. 3:**
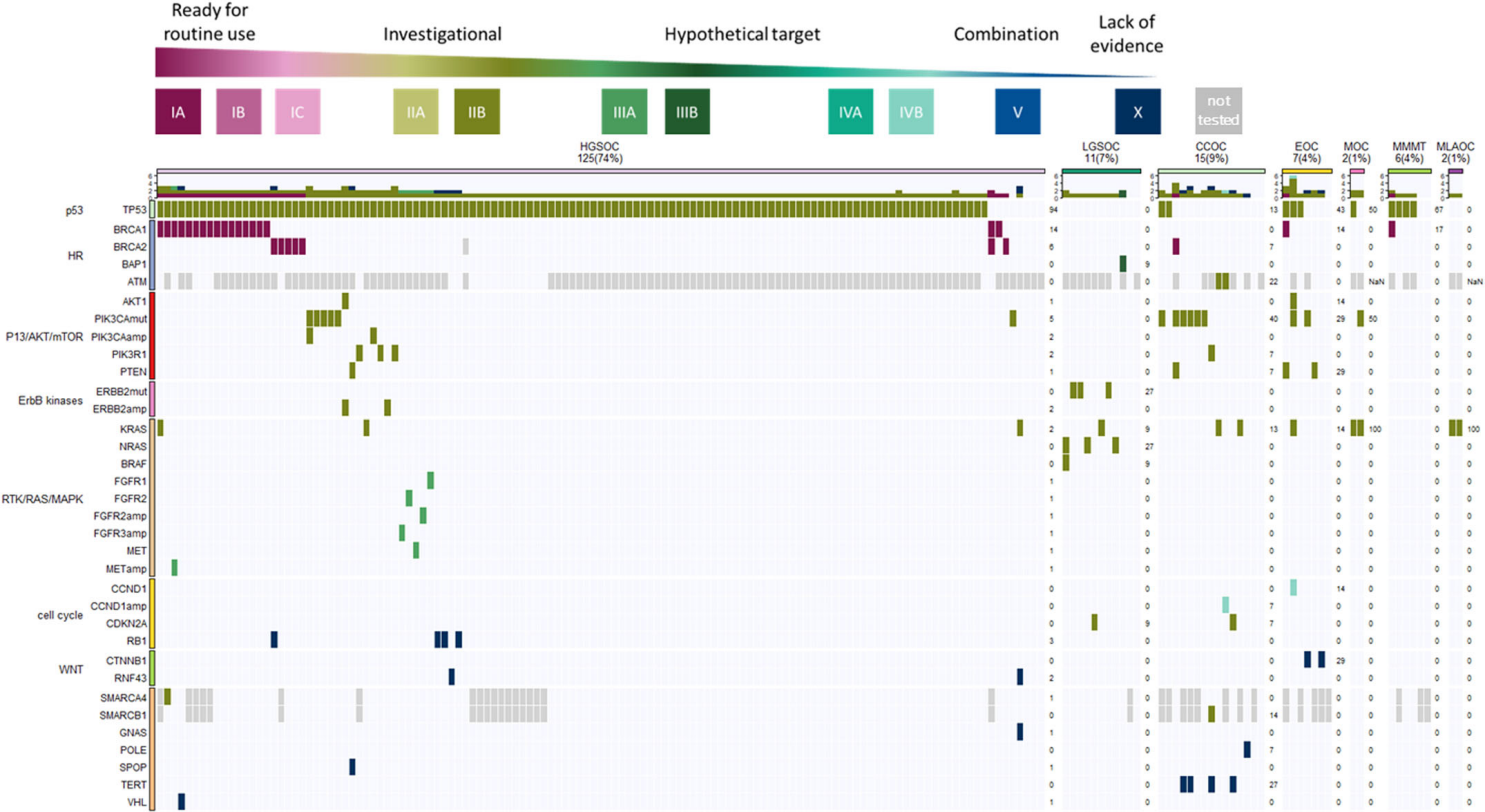
Frequencies of actionable genetic variants per gene across ovarian histotypes. Drug-target matches were annotated based on their clinical actionability according to ESCAT and patients were assigned to the highest level of actionability. The oncoplot shows a summary of the genes affected (rows) in individual patients (columns). Genes are grouped by pathways and patients are grouped according to histotype.

We investigated if specific molecular pathways were involved per histotype (Fig. 3). The p53 signaling pathway was most frequently altered regardless of the histotype (127/168). Almost all HGSOC tumors (117/125) had genetic variants in the TP53 signaling pathway. Additionally, the HR (21/125), followed by PI3K/AKT/MTOR (11/125), RTK/RAS/MAPK (9/125), cell cycle (4/154), ERBB2 (2/125), and WNT (2/125) pathways were altered in this subtype. In contrast, nearly half of CCOC tumors (7/15) displayed an aberrant P13/AKT/MTOR pathway, while TP53 (2/15), HR (2/15), RTK/RAS/MAPK (2/15), and cell cycle (2/15) were less frequently altered. In LGSOC tumors alterations in the RTK/RAS/MAPK (4/11) and ERBB2 (3/11) pathways were most prevalent. The HR and cell cycle pathways were each affected in only one LGSOC tumors each. Four out of seven EOC tumors displayed an aberrant PI3K/AKT/MTOR signaling pathway, besides the TP53 (3/7), WNT (2/7), and cell cycle (1/7) pathways. Four out of six adenocarcinomas had an altered TP53 pathway, and in one a pathogenic variant in the HR pathway was detected. The RTK/RAS/MAPK pathway was affected in both mucinous tumors that underwent multi-gene panel testing, in addition to the PI3K/AKT/MTOR pathway or TP53 pathway. Both mesonephric-like adenocarcinomas were altered in the RTK/RAS/MAPK pathway but no other pathways were affected. Although there are some trends, no significantly enriched pathways between the subgroups could be obtained, as the number of some histotypes is rather small.

### Classification of genetic variants using ESCAT

To determine the clinical utility of the variants identified by the multi-gene cancer panel, ESCAT scores were assigned for all genetic variants detected in 168 patients who underwent multi-gene panel testing. A comprehensive list of all clinical evidence leading to ESCAT ranking is described in Supplementary Table 3. In 156/168 (93%) OC tumors, at least one actionable alteration was detected. Noteworthy, only 17% (28/168) were classified as ESCAT tier-I (*BRCA1* and *BRCA2*). Nearly all patients (151/168) had an ESCAT tier-II classified variant, most frequently in *TP53* (127/168), followed by *PIK3CA* (15/168) and *KRAS* (11/168). Rare tier-II variants, affecting <3% of the OC tumors include: *ATM, AKT1, PIK3R1, CDKN2A, PTEN, ERBB2, NRAS, BRAF, SMARCA4*, and *SMARCB1*. All other variants were scored as ESCAT tier-IIIA, tier-IIIB, tier-IV or tier-X. The oncoplot in Fig. 3 provides a detailed overview of all genetic variants affecting individual patients across different histotypes. ESCAT-scores correlated with the age of the patient are summarized in a bar chart in Supplementary Fig. 1.

## Discussion

This study unraveled the genomic landscape from a unique cohort of 351 Belgian OC patients across different histotypes using real-world data. Rapid evolvement of next-generation sequencing currently allows the medical community to produce a myriad of genetic information; the clinical utility, however, is not always apparent. Here, we demonstrated that a cancer gene panel offers a focused approach allowing the medical community to capture the most relevant genetic variants and that scoring their clinical utility is feasible.

The first objective of this study was to give an overview of the *BRCA1/2* mutational status in epithelial OC. In total, eleven (4%) patients were found to have a germline, and 39 (11%) had a somatic (likely) pathogenic *BRCA1/2* variant. Compared to other reports from European populations (15–20%), the frequency of *BRCA1/2* variants in our cohort (14%) is at the lower limit^11,18,19^. As expected, the majority (94%) of *BRCA1/2* variants were detected in HGSOC tumors. Only two out of fifteen CCOC tumors, one out of fourteen EOC and one out of eleven MMMT had a *BRCA1/2* variant. Due to relatively small numbers of rare histotypes in our study and in literature, it is hard to compare our findings with those of others. Overall, our prevalence of *BRCA1/2* variants appears to be somewhat lower, except for the CCOC histotype. Similar to other reports, no *BRCA1/2* variants were found in MOC tumors^20^. Another difference is the higher occurrence of somatic versus germline variants in our cohort. A recent review reported that 13%–21% of epithelial OC patients have germline *BRCA1/2* variants and only 6% harbor somatic *BRCA1/2* variants^18^. Historically, *BRCA1/2* testing was biased towards patients with a family history of breast/ovarian cancer and focused on patients with early-onset disease, which may explain higher mutation detection rates in other studies^11^. However, we cannot exclude a bias in referral for our cohort, as tumors from patients with known germline variants may not have been referred for tumor testing, especially before 2019.

As the majority of the *BRCA1/2* variants are of somatic origin, the mutation spectrum is more diverse than previously described for germline variants in the Belgian OC population. In 2004, Claes et al. reported six major recurrent *BRCA1/2* variants accounting for 60% of all identified variants in a Belgian cohort. Only two of these recurrent germline variants (*BRCA2* c.6275_6276del & c.8904del) were found in two patients of our cohort^21^.

About 50% of all somatic *BRCA1/2* variants, 50% of *BRCA1* and 25% of *BRCA2* germline variants were detected in the OCCRs, displaying only a small enrichment of variants in the OC cluster regions. Although, the RING-domain is associated with a lower OC risk compared to breast cancer, still one germline and six somatic *BRCA1* variants were detected in this region. An exploratory analysis of the PAOLA-1 trial suggested that the location of mutation in *BRCA1/2* could influence the magnitude of benefit from platinum salts and/or olaparib (plus bevacizumab): patients with mutations in the DBD region of BRCA2 might be extremely sensitive to platinum, whereas those with mutations in the DBD region of BRCA1 might be less sensitive to platinum but extremely sensitive to olaparib plus bevacizumab. Currently, the biological mechanisms underlying domain-related sensitivity to PARPi are unknown. However, if this intriguing finding could be confirmed in independent studies, treatments may not only be guided by the mutated gene but also by the location of the variant^22^.

The second objective of this study was to evaluate the prevalence of additional actionable genetic variants in epithelial OC beyond *BRCA1/2*. In 157/168 OCs that underwent multi-gene panel testing, 242 (likely) pathogenic alterations were detected. These variants were ranked according to ESCAT into six levels of evidence ranging from ‘ready for routine use’ (tier-I) to ‘lack of evidence’ (tier-X). Tier-I variants (*BRCA1* and *BRCA2*) were found in nearly 17% of the OC tumors. Several randomized clinical trials have demonstrated improved oncological outcomes with PARPi maintenance treatment over standard of care in newly diagnosed OC patients with *BRCA1/2* genetic variants, leading to EMA and FDA approvals^8,10,22^. OC patients harboring tier-IIB variants can be enrolled in several ongoing clinical studies that test the efficacy of a drug matched to genetic variants, without data being available on overall or disease-free survival. Genes in this category include: *TP53, ATM, AKT1, PIK3CA, PIK3R1, PTEN, ERBB2, KRAS, NRAS* and *BRAF*. One notable example is the BOUQUET trial (NCT04931342) that evaluates the efficacy and safety of biomarker-driven therapy in patients with platinum-resistant OC of a rare (non-HGSOC) histotype. In BOUQUET, *PIK3CA*, *AKT1*, *PTEN*, *ERBB2*, *KRAS*, *NRAS* and *BRAF*, are being used as biomarkers to plan individualized study treatment^23,24^. Other large ongoing studies on the effectiveness of targeted anti-cancer drugs and immunotherapy, in patients where the tumor is known to have specific genetic variants are NCI-MATCH (NCT02465060), DRUP (NCT02925234) and TAPUR (NCT02693535)^25–28^. Concerning tier-IIIA, OC tumors with *FGFR1/2/3* pathogenic variants could be treated with FGFR-inhibitors. These are classified as tier-IA in other tumor types (like urothelial carcinoma and biliary tract cancer^14^) and the ongoing NCI-MATCH trial is investigating, amongst others, the efficacy of FGFRi in additional tumor types^29^. However, to date no supportive clinical data is yet available in OC. *ERBB2* amplified tumors are matched to anti-HER2 antibody, trastuzumab, treatment and are classified as tier I-A in breast cancer and are currently investigated in multiple clinical trials in OC patients (e.g. DESTINY: phase 2. NCT04482309). MET inhibitors have shown significant benefit for treatment in *MET* amplified lung cancers (tier-II), however, data for OC is currently still lacking. *BAP1* was categorized as tier-IIIB, as this is a protein that interacts with *BRCA1/2* to mediate homologous recombination during DNA repair. Testing PARPis in patients with a deficient *BAP1* protein would be conceptually reasonable. Several clinical trials targeting *BAP1* alterations in multiple cancer types (not OC) are ongoing (e.g. NCT03207347, NCT04666740, NCT03654833, NCT02925234). Two groups investigated whether silencing of Cyclin D1 (*CCND1*) could lead to a BRCAm-like phenotype and thus improve the efficacy of PARPis. Micro-RNA and short hairpin-RNA were used in vitro and in vivo in mice to downregulate *CCND1* resulting in a substantial benefit in OC management (tier-IVA)^30,31^. Other genes like, *POLE*, *RB1*, *TERT*, *GNAS*, *CTNNB1*, *RNF43* and *SPOP* have prognostic values or are frequently altered/upregulated in certain histologic subtypes, but are currently not considered as targets matched to specific treatments (tier-X)^32–38^.

To our knowledge, the present study is the most comprehensive study classifying alterations in a large epithelial OC cohort using ESCAT. Previously, Lapke et al. reported actionable variants in a smaller group of OCs (*n* = 85). In their study, eligibility for targeted therapy was determined by a pathway-based approach; however, no classification according to degrees of clinical evidence was applied^39^. ESCAT classification has been applied in other cancer types, including head and neck cancer, breast cancer and tumor-agnostic studies; these reported that implementation of ESCAT for clinical-decision making is feasible and beneficial for personalized treatment^14–17,40–44^. ESCAT scoring in our cohort only identified a limited number of patients (17%) with tier-I genetic variants; however, tier-II actionable variants were detected in 90% of the patients. This implies that only a small proportion of reported genetic variants have a direct impact on treatment decisions. Nevertheless, many patients are potentially eligible to specific clinical trials tailored to their cancer’s genome and could benefit from drugs targeting a tier-II variant. We hypothesize that one reason why OC is lagging behind other cancer types towards targeted treatments is conventional trial design. Trials in which all patients with a specific cancer type receive the same treatment limit the possible benefits of precision oncology. From our data it is clear that rare histologic subtypes have distinct molecular features that are possibly actionable. Since the most common histotype is HGSOC, it is often the only subtype included in clinical trials. Recently, clinical research has evolved from using cancer type-centered to biomarker-directed and histology-specific trials. These ‘master protocol’ designs are tailored to molecular profiles of the patients.

To improve the concept of personalized treatment, the strategy should be implemented earlier in the course of the disease and tumors should have comprehensive tumor profiling at each stage of the disease^45,46^. Another major challenge for oncologists remains how to integrate patients’ biomarker profiles into therapeutic decision-making as literature is complex, rapidly evolving, and voluminous. Next, local drug access is also critical for clinicians seeking therapeutic options for their patients. To aid clinicians, several knowledge bases have been developed. Recently, a curated version specific to the Australian healthcare setting was developed: TOPOGRAPH—Therapy-oriented precision oncology guidelines for recommendation of anticancer pharmaceuticals^47^. But utility studies on such an electronic compendium with respect to treatment recommendations are warranted.

Strengths of our study include a relatively large sample size that covered a large spectrum of histotypes in OC, the participation of a single expert center with uniform management procedures and NGS testing, including variant interpretation, a small proportion of patients excluded for analysis, full access to all relevant pathology specimens for rigorous review, and the extensive literature search for assessing strength of evidence using ESCAT. However, several limitations should be considered when interpreting the results of this study. First, the retrospective and anonymized design precluded the collection of additional data or samples types. Therefore, we could neither assess patients’ survival time, nor determine whether or not patients received matched drugs to genetic variants. Second, some concerns may be raised that genomic instability evaluating the consequence of HRD beyond *BRCAm* (e.g., loss of heterozygosity, telomeric allelic imbalance and/or large-scale state transitions) was not assessed in our study. Although our panel did contain two genes involved in HRD beyond *BRCA1/2* (i.e.*, BAP1* and *ATM*), it has been demonstrated that mutations in non-*BRCA* homologous recombination repair genes (HRRm) did not predict benefit from olaparib (a PARPi) plus bevacizumab in PAOLA-1; hence, non-*BRCA* HRRm in multigene panels do not substitute for HRD determined by genomic instability testing (and *BRCA* mutation status)^48^. Third, clear deletions or amplifications are picked up by coverage analysis but smaller exon-spanning deletions or duplications, gene fusions, mutations in regulatory sequences or deep-intronic mutations could not be detected by the applied molecular analysis. Bearing this in mind, some highly druggable targets are missed (e.g. *NTRK* fusions)^49^. Similarly (fourth), our methods did not yet assess microsatellite instability and tumor mutational burden (too small panel size around 100 kb), although both have been shown to be predictive biomarkers of immunotherapy in several solid tumors (albeit with limited evidence in OC). Fifth, the ESCAT is a validated and well-respected instrument; however, tumor heterogeneity (i.e., spatial differences) and evolutionary dynamics of the cancer genome (i.e., temporal differences) may hinder the more extensive use of molecular profiling-based strategies. Sixth, as our cohort comprised mainly Caucasian women, representative of the Flemish population, validations in other ethnicities are warranted. Therefore, in a similar prospective study design most of these limitations will disappear. Other limitations like MSI status and tools for detecting exon-spanning deletions/amplifications should be tested and validated. Spatial differences and temporal differences could be addressed using cfDNA mutational analysis at different time points during a patients’ treatment course, although, this will cost more and implementing this into standard clinical practice may take some time.

In conclusion, we unveiled a diverse landscape of potentially actionable genetic variants in this histologically and genetically well-defined cohort of OC patients. Unfortunately, directly actionable (ESCAT tier-I) variants accounted only for a small proportion. Multiple biomarker-driven clinical trials recruiting OC patients are currently ongoing; these trials generate information that could ultimately lead to the dawn of precision oncology in OC patients.

## Methods

### Patients and samples

This retrospective cohort study included 358 patients diagnosed with OC between January 2014 and December 2022 in 28 hospitals across East-Flandres and West-Flandres. These patients underwent molecular testing of blood and/or tumor samples at the Center for Medical Genetics Ghent (Ghent University Hospital, Belgium). The study was approved by the ethics committee of Ghent University Hospital (ONZ-2023-0389); the need for informed consent of patients was waived because of the retrospective nature of the study and anonymized data was transferred to the investigators. The study was conducted in accordance with the Declaration of Helsinki and followed the ‘Strengthening the Reporting of Observational Studies in Epidemiology’ (STROBE) guideline. Histopathologic information and molecular sequencing data were extracted from the institutional electronic health records. A gynecological pathologist (KV) reviewed all available tumor slides (using both HE and immunohistochemistry) in a blind manner to classify them into distinct ovarian cancer histotypes: high-grade serous ovarian cancer (HGSOC), low-grade serous ovarian cancer (LGSOC), clear cell carcinoma of the ovaries (CCOC), endometrioid ovarian cancer (EOC), mucinous ovarian cancer (MOC), mixed malignant mullerian tumor (MMMT) and mesonephric-like adenocarcinoma of the ovary (MLAOC). All borderline or non-epithelial tumors were excluded for further analysis.

### Molecular profiling

Tumor samples obtained before January 2019 were only examined for somatic genetic variants in *BRCA1* and *BRCA2* using the PCR-based BRCA Tumor Mastr Plus Dx kit (Multiplicom). In parallel, germline testing was performed for *BRCA1*, *BRCA2*, *TP53* and *PALB2* using an in-house developed amplicon-based target enrichment method, followed by next generation sequencing (Miseq, Illumina) as previously described^50^. Starting from 2019, a capture-based approach was applied for target enrichment. A detailed description of the methods can be found in Supplementary Methods 1. Both tumor and blood samples were examined using a customized cancer gene panel that has been updated over time (all panel versions can be found in Supplementary Tables 4–6). Only variants that were classified as pathogenic or likely pathogenic were considered for further analysis.

### Functional domains BRCA1 and BRCA2

Functional domains were defined as:

BRCA1: RING (Really Interesting New Gene) domain: amino acids (AA) 8–96, DBD (DNA-Binding Domain): AA 452–1092, BRCT (BRCA1 C-Terminal) domain: AA 1646-1736 and AA 1760-1855.

BRCA2: RAD51-BD (RAD51-Binding Domain): AA 900-2000, DBD: AA 2459-3190.

The Ovarian Cancer Cluster Regions (OCCRs) were defined for BRCA1: AA 460-1354; and for BRCA2: OCCR1 - AA 1083-1894 and OCCR2- AA 2215-2490^20,51^.

### Level of actionability

Genetic variants were ranked according to the ESMO Scale for Clinical Actionability of molecular Targets (ESCAT) into six levels of evidence ranging from ‘ready for routine use’ (tier-I) to ‘lack of evidence’ (tier-X), previously described by Mateo et al.^13^. To rank the specific variants, the results of clinical trials (ClinicalTrials.gov) were summarized and classified based on several characteristics including prospectiveness, randomization, and availability of results. When no clinical trials in OC were available, basket trials or trials in other cancer types were examined. All listed drug-target matches were investigated for FDA or EMA approvals and for treatment recommendations by NCCN. OncoKB and publications which already describe ESCAT scoring systems in other cancer types were used to propose a score in case of doubt about drug-target matches^13–17,52–54^. The proposed ranking was validated by the co-authoring medical oncologists, pathologists and clinical laboratory geneticists until consensus. Patients with multiple genetic variants were ranked by the highest level of evidence. A comprehensive report of all genetic variants and matched drugs accompanied with clinical evidence and ESCAT ranking is included in Supplementary Table 3^13^.

### Signaling pathways

For the most frequently mutated genes the canonical pathways in which they are playing a role, were determined. Five signaling pathways were previously curated by TCGA PanCancer Atlas^54^: (1) cell cycle pathway (*CCND1, CDKN2A, RB1)*, (2) RTK/RAS/MAPK pathway (*KRAS, NRAS, BRAF, FGFR1, FGFR2, FGFR3, MET)*, (3) p53 pathway *(TP53)*, (4) PI3K/AKT/mTOR pathway (*AKT1, PIK3CA, PIK3R1, PTEN)*, (5) WNT pathway (*CTNNB1, RNF43)*. Two additional pathways included the HR pathway (*BRCA1, BRCA2, BAP1, ATM*) and ErbB kinases (*ERBB2*). Seven additional genes with at least one genetic variant could not be assigned to any of these seven pathways (*SMARCA4, SMARCB1, GNAS, POLE, SPOP, TERT, VHL*).

### Statistical analysis

All analyses and visualizations were performed using Excel version 2307 and R Studio version R4.3.1 (R program for Statistical Computing). To summarize all actionable variants for the individual patients, an oncoprint was generated in R Studio using the Complex Heatmap package. Patients were grouped according to histotype; genes were grouped in their respective pathway. The lollipop plot for *BRCA1* and *BRCA2* variants was adapted from cBioportal (genome build GRCh38).

### Reporting summary

Further information on research design is available in the Nature Research Reporting Summary linked to this article.

### Supplementary information

Supplemental Material

REPORTING SUMMARY

## Data Availability

All data analyzed in this study are included in the published article and its supplementary information files. Raw data are available from the corresponding author. Qualified researchers can apply for access to these datasets via a collaboration or data usage agreement. The data are not publicly available due to privacy and ethical restrictions.

## Code availability

Relevant R script codes are available from the corresponding author upon request.
