# Supplementary material for: Identification of potentially actionable genetic variants in epithelial ovarian cancer: a retrospective cohort study
Source: NPJ Precis Oncol. 2024 Mar 22;8:71. doi: 10.1038/s41698-024-00565-2 (PMC10959961; doi:10.1038/s41698-024-00565-2)
Supplement: Supplementary file 1 — Supplemental Material [file 41698_2024_565_MOESM1_ESM.pdf]

Supplementary Table 1 – overview of somatic and germline (likely) pathogenic *BRCA1/2* variants identified in ovarian cancer patients between 2014 and 2022, with indication of their location in a functional domain and/or ovarian cancer cluster region

	Patient n°	Histotype	C-notation	P-notation	S/G	Functional Domain[1]	Cluster Region[2]
BRCA1	10	HGSOC	c.3001G>T	p.(Glu1001Ter)	somatic	DBD	OCCR
	19	HGSOC	c.135-2A>G	p.(?)	somatic	RING	
	22	HGSOC	c.5194-1G>T	P. (?)	somatic	BRCT	
	41	HGSOC	c.2603C>G	p.(Ser868Ter)	germline	DBD	OCCR
	44	HGSOC	c.134+3A>C	p.(?)	germline	RING	
	51	HGSOC	c.2223del	p.(Asn742IlefsTer11)	somatic	DBD	OCCR
	64	HGSOC	c.4065_4068del	p.(Asn1355LysfsTer10)	somatic		
	65	HGSOC	c.2507_2508del	p.(Glu836GlyfsTer2)	germline	DBD	OCCR
	67	MMMT	c.869T>A	p.(Leu290Ter)	somatic		
	81	HGSOC	c.68_80+6del	p.(?)	somatic	RING	
	92	HGSOC	c.1816_1826del	p.(Pro606Ter)	somatic	DBD	OCCR
	95	HGSOC	c.4576G>T	p.(Glu1526Ter)	somatic		
	117	HGSOC	c.1395T>A	p.(Tyr465Ter)	somatic	DBD	OCCR
	133	HGSOC	c.5309G>T	p.(Gly1770Val)	germline	BRCT	
	137	HGSOC	c.3362del	p.(Asn1121fs)	somatic	DBD	
	173	HGSOC	c.3748G>T	p.(Glu1250Ter)	somatic		OCCR
	180	HGSOC	c.2216_2217del	p.(Lys739SerfsTer3)	somatic	DBD	OCCR
	187	HGSOC	c.(134+1_135-1)_ (301+1_302-1)del	p.(del. Exon 5&6)	germline	RING	
	200	HGSOC	c.66dup	p.(Glu23ArgfsTer18)	somatic	RING	
	207	HGSOC	c.3788T>A	p.(Leu1263Ter)	somatic		OCCR
	213	CCOC	c.181T>G	p.(Cys61Gly)	somatic	RING	
	227	HGSOC	c.2745dup	p.(Asn916Ter)	somatic	DBD	OCCR
	231	HGSOC	c.1961dup	p.(Tyr655)	somatic	DBD	OCCR
	239	HGSOC	c.981_982del	p.(Cys328Ter)	somatic		
	243	HGSOC	c.4327C>T	p.(Arg1443Ter)	somatic		
	271	HGSOC	c.3520_3821del	p.(Ser1174AsnfsTer12)	somatic		OCCR
	287	HGSOC	c.5074+1G>T	p.(?)	somatic	BRCT	
	297	HGSOC	c.2118del	p.(Gly707ValfsTer29)	somatic	DBD	OCCR
	298	EOC	c.2507_2508del	p.(Glu836GlyfsTer2)	somatic	DBD	OCCR
	305	HGSOC	c.3598C>T	p.Gln1200Ter)	somatic		OCCR
	306	HGSOC	c.212G>T	p.(Arg71Met)	somatic	RING	
	323	HGSOC	c.3481_3491del	p.(Glu1161PhefsTer3)	germline		OCCR
	348	HGSOC	c.4658T>A	p.(Leu1553Ter)	somatic		
BRCA2	69	CCOC	c.6244G>T	p.(Glu2082Ter)	somatic		
	86	HGSOC	c.8184del	p.(Lys2729ArgfsTer4)	somatic	DBD	
	87	HGSOC	c.1389_1390del	p.(Thr463fs)	somatic		
	92	HGSOC	c.658_659del	p.(Val220IlefsTer4)	germline		
	112	HGSOC	c.9405_9420del	p.(Leu3136GlufsTer22)	somatic	DBD	
	151	HGSOC	c.2175dup	p.(Val726SerfsTer25)	somatic		
	170	HGSOC	c.5222_5223del	Ser1741LysfsTer3	somatic	RAD51-BD	OCCR1
	189	HGSOC	c.3599_3600del	p.(Cys1200Ter)	somatic	RAD51-BD	OCCR1
	212	HGSOC	c.9294C>G	p.(Tyr3098Ter)	germline	DBD	
	265	HGSOC	c.8243G>A	p.(Gly2748Asp)	germline	DBD	
	267	HGSOC	c.4689G>A	p.(Trp1563Ter)	somatic	RAD51-BD	OCCR1
	275	HGSOC	c.8904del	p.(Val2969CysfsTer7)	germline	DBD	
	288	HGSOC	c.6275_6276del	p.(Leu2092ProfsTer7)	germline		
	302	HGSOC	c.5073del	p.(Lys1691AsnfsTer15)	somatic	RAD51-BD	OCCR1
	308	HGSOC	c.9097dup	p.(Thr3033AsnfsTer11)	somatic	DBD	
	343	HGSOC	c.8021dup	p.(Ile2675AspfsTer6)	somatic	DBD	
	351	HGSOC	c.6952C>T	p.(Arg2318Ter)	somatic		OCCR2

[1] the functional domains were defined as: *BRCA1*, RING domain: amino acids (AA) 8-96; DBD: AA 452-1092; BRCT: AA 1646-1736 and 1760-1855; *BRCA2*, RAD51-BD: AA 900-2000; DBD: AA 2459-3190 [19]

[2] the ovarian cancer cluster regions were defined as: *BRCA1,* OCCR amino acids (AA)460-1354. *BRCA2*, OCCR1: AA 1083-1894 and OCCR2: AA 2215-2490[20]

Supplementary Table 2

patient	age	TCP (%)	histological subtype	Gene panel	gene	c-notation	protein	VAF (%)	ESCAT score	confirmed germline
1	69	80	HGSOC	1	*TP53*	c.659A>G	p.(Tyr220Cys)	67	IIB	
4	65	20	HGSOC	1	*TP53*	c.271_272delinsA	p.(Trp91SerfsTer32)	14	IIB	
7	67	50	HGSOC	1	*TP53*	c.298del	p.(Gln100ArgfsTer23)	42	IIB	
11	73	80	HGSOC	1	*TP53*	c.673-2A>G	p.(?)	50	IIB	
13	67	80	HGSOC	1	*TP53*	c.715A>G	p.(Asn239Asp)	57	IIB	
14	71	80	HGSOC	1	*FGFR3*	amplificatie			IIIA	
			HGSOC	1	*TP53*	c.659A>G	p.(Tyr220Cys)	72	IIB	
15	43	50	LGSOC	1	*ERBB2*	c.2314_2325dup	p.(Tyr772_Ala775dup)	37	IIB	
16	29	30	LGSOC	1	*ERBB2*	c.2313_2324dup	p.(Tyr772_Ala775dup)	10	IIB	
19	62	75	HGSOC	1	*BRCA1*	c.135-2A>G	p.(?)	38	IA	
			HGSOC	1	*TP53*	c.661G>T	p.(Glu221Ter)	43	IIB	
24	65	75	HGSOC	1	*TP53*	c.524G>A		91	IIB	
26	62	40	CCOC	2	*KRAS*	c.35G>A	p.(Gly12Asp)	29	IIB	
31	67	30	HGSOC	1	*KRAS*	c.35G>A	p.(Gly12Asp)	35	IIB	
			HGSOC	1	*GNAS*	c.601C>T	p.(Arg201Cys)	14	X	
			HGSOC	1	*RNF43*	c.847C>T	p.(Gln283Ter)	42	X	
39	85	50	HGSOC	1	*TP53*	c.916C>T	p.(Arg306Ter)	13	IIB	
43	72	40	HGSOC	1	*TP53*	c.713G>A	p.(Cys238Tyr)	12	IIB	
			HGSOC	1	*PIK3CA*	c.1633G>A	p.(Glu545Lys)	5	IIB	
45	60	80	EOC	2	*CTNNB1*	c.98C>T	p.(Ser33Phe)	44,9	X	
			EOC	2	*ATM*	c.7629+1G>A	p.?	5	IIB	
47	71	80	HGSOC	1	*TP53*	c.550_551del	p.(Asp184Ter)	24	IIB	
49	85	20	HGSOC	2	*TP53*	c.524G>A	p.(Arg175His)	46	IIB	
51	69	60	HGSOC	1	*TP53*	c.487T>C	p.(Tyr163His)	53	IIB	
			HGSOC	1	*BRCA1*	c.2223del	p.(Asn742IlefsTer11)	50	IA	
54	64	30	CCOC	1	*TERT*	c.250C>T	p.(?)	11	X	
			CCOC	1	*CDKN2A*	c.238C>T	p.(Arg80Ter)	3	IIB	
55	55	60	HGSOC	1	*KRAS*	c.35G>T	p.(Gly12Val)	10	IIB	
			HGSOC	1	*TP53*	c.378C>A	p.(Tyr126Ter)	8	IIB	

56	68	40	HGSOC	1	*TP53*	c.1010G>T	p.(Arg337Leu)	28	IIB	
			HGSOC	1	*PTEN*	c.445C>T	p.(Gln149Ter)	18	IIB	
			HGSOC	1	*SPOP*	c.362G>A	p.(Arg121Gln)	13	X	
57	72	60	HGSOC	1	*TP53*	c.536A>G	p.(His179Arg)	70	IIB	
59	70	50	HGSOC	1	*TP53*	c.737T>G	p.(Met246Arg)	59	IIB	
61	55	80	MMMT	1	*TP53*	c.695T>C	p.(Ile232Thr)	81	IIB	
64	75	90	HGSOC	2	*BRCA1*	c.4065_4068del	p.(Asn1355LysfsTer10)	54	IA	
			HGSOC	2	*TP53*	c.844C>T	p.(Arg282Trp)	61	IIB	
			HGSOC	2	*KRAS*	c.35G>C	p.(Gly12Ala)	15	IIB	
65	56	70	HGSOC	1	*BRCA1*	c.2507_2508del	p.(Glu836GlyfsTer2)	77	IA	x
			HGSOC	1	*TP53*	c.817C>T	p.(Arg273Cys)	41	IIB	
67	53	60	MMMT	1	*BRCA1*	c.869T>A	p.(Leu290Ter)	30	IA	
			MMMT	1	*TP53*	c.306del	p.(Tyr103ThrfsTer20)	33,0	IIB	
68	74	50	HGSOC	1	*TP53*	c.497C>G	p.(Ser166Ter)	50,0	IIB	
69	46	80	CCOC	1	*BRCA2*	c.6244G>T	p.(Glu2082Ter)	28,0	IA	
			CCOC	1	*PIK3CA*	c.1637A>G	p.(Gln546Arg)	31,0	IIB	
			CCOC	1	*PTEN*	c.388C>T	p.(Arg130Ter)	32,0	IIB	
			CCOC	1	*PTEN*	c.202T>C	p.(Tyr68His)	24,0	IIB	
72	64	30	HGSOC	1	*TP53*	c.686_687del	p.(Cys229TyrfsTer10)	23,0	IIB	
73	70	30	HGSOC	1	*TP53*	c.818G>A	p.(Arg273His)	12,0	IIB	
74	63	60	HGSOC	1	*TP53*	c.422G>A	p.(Cys141Tyr)	64,0	IIB	
79	48	40	HGSOC	2	*TP53*	c.132dup	p.(Leu45AlafsTer7)	40	IIB	
81	65	60	HGSOC	1	*TP53*	c.1048dup	p.(Leu350ProfsTer32)	35	IIB	
			HGSOC	1	*BRCA1*	c.68_80+6del	p.?	39	IA	
82	75	80	HGSOC	1	*TP53*	c.488A>G	p.(Tyr163Cys)	92	IIB	
87	61	80	HGSOC	1	*BRCA2*	c.1389_1390del	p.(Val464GlyfsTer3)	81	IA	
			HGSOC	1	*TP53*	c.818G>A	p.(Arg273His)	75	IIB	
			HGSOC	1	*RB1*	c.111_137+54del	p.(?	51	X	
92	75	80	HGSOC	2	*BRCA2*	c.658_659del	p.(Val220IlefsTer4)	79	IA	x
			HGSOC	2	*BRCA1*	c.1816_1826del	p.(Pro606Ter)	32	IA	
100	77	80	HGSOC	1	*TP53*	c.535C>T	p.(His179Tyr)	45	IIB	
101	71	10	HGSOC	1	*PIK3CA*	c.1258T>C	p.(Cys420Arg)	4	IIB	
			HGSOC	1	*TP53*	c.128del	p.(Leu43Ter)	3	IIB	

102	86	70	MLAOC	1	*KRAS*	c.35G>C	p.(Gly12Ala)	86	IIB	
103	68	60	CCOC	2	*TERT*	c.-57 A>C	p. C228T	21	X	
			CCOC	2	*PIK3CA*	c.3140A>G	p.(His1047Arg)	19	IIB	
111	77	60	HGSOC	2	*TP53*	c.713G>A	p.(Cys238Tyr)	57	IIB	
114	63	20	EOC	2	*PTEN*	c.968dup	p.(Asn323LysfsTer2)	29	IIB	
			EOC	2	*PTEN*	c.801+1G>T	p.?	30	IIB	
115	71	40	HGSOC	2	*TP53*	c.722C>T	p.(Ser241Phe)	28	IIB	
			HGSOC	2	*PIK3R1*	c.2149_2150del	p.(Pro717SerfsTer23)	18	IIB	
118	82	70	HGSOC	1	*TP53*	c.524G>A	p.(Arg175His)	73	IIB	
124	59	60	HGSOC	1	*TP53*	c.734G>A	p.(Gly245Asp)	50	IIB	
128	51	60	MOC	1	*KRAS*	c.35G>A	p.(Gly12Asp)	26	IIB	
			MOC	1	*TP53*	c.524G>A	p.(Arg175His)	41	IIB	
132	65	90	HGSOC	1	*TP53*	c.514G>T	p.(Val172Phe)	39	IIB	
136	72	80	MMMT	2	*TP53*	c.743G>A	p.(Arg248Gln)	56	IIB	
138	62	60	HGSOC	1	*TP53*	c.832C>T	p.(Pro278Ser)	57	IIB	
141	66	40	HGSOC	1	*TP53*	c.586_588del	p.(Arg196del)	18	IIB	
			HGSOC	1	*PIK3CA*	c.317G>T	p.(Gly106Val)	17	IIB	
142	62	80	HGSOC	1	*TP53*	c.329G>T	p.(Arg110Leu)	65	IIB	
			HGSOC	1	*FGFR2*	c.758C>G	p.(Pro253Arg)	36	IIIA	
144	73	40	HGSOC	1	*TP53*	c.637C>T	p.(Arg213Ter)	26	IIB	
145	36	50	EOC	2	*TP53*	c.493C>T	p.(Gln165Ter)	61	IIB	
146	63	50	HGSOC	1	*TP53*	c.824G>A	p.(Cys275Tyr)	21	IIB	
147	77	80	HGSOC	1	*TP53*	c.137_165del	p.(Ser46Ter)	41	IIB	
150	44	60	CCOC	2	*TERT*	c.-124C>T	p.(?)	28	X	
			CCOC	2	*PIK3CA*	c.3140A>G	p.(His1047Arg)	24	IIB	
			CCOC	2	*PIK3CA*	c.1357G>C	p.(Glu453Gln)	24	IIB	
151	77	15	HGSOC	1	*BRCA2*	c.2175dup	p.(Val726SerfsTer25)	7	IA	
			HGSOC	1	*TP53*	c.488A>G	p.(Tyr163Cys)	14	IIB	
153	65	70	LGSOC	1	*NRAS*	c.182A>G	p.(Gln61Arg)	40	IIB	
155	74	40	LGSOC	1	*CDKN2A*	c.387C>A	p.(Tyr129Ter)	19	IIB	
156	82	80	HGSOC	1	*TP53*	c.581T>G	p.(Leu194Arg)	83	IIB	
158	59	70	HGSOC	1	*TP53*	c.624_627del	p.(Asp208GlufsTer38)	49	IIB	
160	69	60	HGSOC	2	*TP53*	c.637C>T	p.(Arg213Ter)	32	IIB	

162	68	20	HGSOC	1	*TP53*	c.223_230del	p.(Pro75SerfsTer71)	7	IIB	
163	39	70	LGSOC	1+2	*NRAS*	c.182A>G	p.(Gln61Arg)	35	IIB	
164	81	20	HGSOC	1	*TP53*	c.818G>A	p.(Arg273His)	13	IIB	
165	63	80	MMMT	1	*TP53*	c.659A>G	p.(Tyr220Cys)	29	IIB	
166	55	60	HGSOC	1	*TP53*	c.743G>A	p.(Arg248Gln)	53	IIB	
167	70	90	HGSOC	1	*TP53*	c.493C>T	p.(Gln165Ter)	83	IIB	
168	65	35	HGSOC	1	*TP53*	c.916C>T	p.(Arg306Ter)	44	IIB	
173	51	60	HGSOC	1	*TP53*	c.725G>C	p.(Cys242Ser)	68	IIB	
			HGSOC	1	*BRCA1*	c.3748G>T	p.(Glu1250Ter)	65	IA	
174	80	25	HGSOC	2	*TP53*	c.97-2del	p.(?)	7	IIB	
179	78	80	HGSOC	2	*TP53*	c.731G>A	p.(Gly244Asp)	74	IIB	
180	85	60	HGSOC	2	*BRCA1*	c.2216_2217del	p.(Lys739SerfsTer3)	63	IA	
			HGSOC	2	*TP53*	c.325T>G	p.(Phe109Val)	47	IIB	
181	65	80	HGSOC	1	*TP53*	c.526T>C	p.(Cys176Arg)	86	IIB	
182	69	40	HGSOC	1	*TP53*	c.445del	p.(Ser149ProfsTer21)	33	IIB	
189	76	60	HGSOC	2	*BRCA2*	c.3599_3600del	p.(Cys1200Ter)	51	IA	
			HGSOC	2	*TP53*	c.339_340dup	p.(Leu114SerfsTer10)	54	IIB	
191	65	70	EOC	1	*KRAS*	c.34G>A	p.(Gly12Ser)	64	IIB	
			EOC	1	*TP53*	c.365_366del	p.(Val122AspfsTer26)	3	IIB	
			EOC	1	*PIK3CA*	c.328_330del	p.(Glu110del)	26	IIB	
			EOC	1	*PIK3CA*	c.112C>T	p.(Arg38Cys)	22	IIB	
			EOC	1	*AKT1*	c.118G>A	p.(Glu40Lys)	35	IIB	
			EOC	1	*CCND1*	c.859C>T	p.(Pro287Ser)	28	IVA	
194	52	70	HGSOC	2	*TP53*	c.637C>T	p.(Arg213Ter)	79	IIB	
196	60	50	HGSOC	1	*TP53*	c.524G>A	p.(Arg175His)	70	IIB	
197	67	80	HGSOC	1	*TP53*	c.991C>T	p.(Gln331Ter)	74	IIB	
200	66	40	HGSOC	1	*BRCA1*	c.66dup	p.(Glu23ArgfsTer18)	76	IA	
207	59	60	HGSOC	1	*BRCA1*	c.3788T>A	p.(Leu1263Ter)	63	IA	
			HGSOC	1	*SMARCA4*	c.3634G>T	p.(Glu1212Ter)	27	IIB	
			HGSOC	1	*TP53*	c.375+5G>T	p.(?)	65	IIB	
210	70	20	HGSOC	1	*TP53*	c.659A>G	p.(Tyr220Cys)	7	IIB	
211	42	50	HGSOC	1	*TP53*	c.994-2A>G	p.(?)	43	IIB	
213	43	40	CCOC	2	*TP53*	c.743G>A	p.(Arg248Gln)	10	IIB	

216	54	50	HGSOC	1	*TP53*	c.706T>C	p.(Tyr236His)	26	IIB	
218	70	60	HGSOC	1	*PIK3CA*	c.1634A>C	p.(Glu545Ala)	75	IIB	
			HGSOC	1	*TP53*	c.844C>T	p.(Arg282Trp)	33	IIB	
			HGSOC	1	*PIK3CA*	amp			IIB	
222	78	30	HGSOC	1	*TP53*	c.396G>T	p.(Lys132Asn)	23	IIB	
223	71	80	LGSOC	1	*NRAS*	c.181C>A	p.(Gln61Lys)	65	IIB	
			LGSOC	1	*BRAF*	c.1391G>T	p.(Gly464Val)	22	IIB	
224	71	50	HGSOC	1	*TP53*	c.758_760del	p.(Thr253del)	34	IIB	
225	75	60	HGSOC	2	*TP53*	c.672G>T	p.(Glu224Asp)	65	IIB	
226	54	10	LGSOC	1	*KRAS*	c.35G>A	p.(Gly12Asp)	16	IIB	
227	66	10	HGSOC	1	*BRCA1*	c.2745dup	p.(Asn916Ter)	15	IA	
			HGSOC	1	*TP53*	c.824G>T	p.(Cys275Phe)	10	IIB	
228	74	30	HGSOC	1	*TP53*	c.808T>C	p.(Phe270Leu)	9	IIB	
231	69	60	HGSOC	2	*BRCA1*	c.1961dup	c.1961dup	57	IA	
			HGSOC	2	*TP53*	c.844C>T	p.(Arg282Trp)	51	IIB	
232	70	60	HGSOC	1	*TP53*	c.841G>A	p.(Asp281Asn)	36	IIB	
234	68	80	LGSOC	1	*ERBB2*	c.2313_2324dup	p.(Tyr772_Ala775dup)	37	IIB	
235	68	70	HGSOC	1	*TP53*	c.711G>A	p.(Met237Ile)	62	IIB	
236	54	80	CCOC	1	*PIK3CA*	c.1636C>G	p.(Gln546Glu)	56	IIB	
			CCOC	1	*PIK3CA*	c.344G>T	p.(Arg115Leu)	58	IIB	
237	73	60	HGSOC	1	*TP53*	c.396G>C	p.(Lys132Asn)	83	IIB	
239	59	70	HGSOC	1	*BRCA1*	c.981_982del	p.(Cys328Ter)	87	IA	
			HGSOC	1	*VHL*	c.241C>T	p.(Pro81Ser)	40	X	
			HGSOC	1	*TP53*	c.742del	p.(Arg248GlyfsTer97)	79	IIB	
244	68	30	HGSOC	1	*TP53*	c.817C>T	p.(Arg273Cys)	46	IIB	
251	78	75	HGSOC	1	*PIK3CA*	c.1633G>A	p.(Glu545Lys)	26	IIB	
			HGSOC	1	*TP53*	c.850A>C	p.(Thr284Pro)	91	IIB	
252	54	60	HGSOC	1	*TP53*	c.887_913delinsCCCA	p.(His296Profs*33)	15	IIB	
			HGSOC	1	*MET*	c.3082G>T	p.(Asp1028Tyr)	17	IIIA	
254	73	70	HGSOC	1	*TP53*	c.659A>G	p.(Tyr220Cys)	71	IIB	
			HGSOC	1	*PIK3CA*	amp			IIB	
257	47	75	EOC	1	*PIK3CA*	c.1624G>A	p.(Glu542Lys)	29	IIB	
			EOC	1	*CTNNB1*	c.110C>G	p.(Ser37Cys)	21	X	

258	69	40	HGSOC	1	*TP53*	c.841G>A	p.(Asp281Asn)	84	IIB	
259	51	70	CCOC	1	*TERT*	c.228C>T	p.(?)	30	X	
			CCOC	1	*PIK3R1*	c.1678G>T	p.(Asp560Tyr)	27	IIB	
			CCOC	1	*SMARCB1*	c.1148G>A	p.(Arg383Gln)	32	IIB	
261	80	75	LGSOC	1	*BAP1*	c.1983+1G>T	p.?	28	IIIB	
			LGSOC	1	*BAP1*	c.281_287del	p.(His94ArgfsTer2)	24	IIIB	
262	78	95	HGSOC	1	*TP53*	c.455dup	p.(Pro153AlafsTer28)	90	IIB	
			HGSOC	1	*FGFR2*	amp			IIIA	
266	58	70	HGSOC	1	*FGFR1*	c.1731C>A	p.(Asn577Lys)	19	IIIA	
			HGSOC	1	*TP53*	c.378C>A	p.(Tyr126Ter)	69	IIB	
267	90	60	HGSOC	1	*BRCA2*	c.4689G>A	p.(Trp1563Ter	69	IA	
			HGSOC	1	*TP53*	c.800G>C	p.(Arg267Pro)	56	IIB	
269	64	60	HGSOC	1	*TP53*	c.376-1G>A	p.(?)	41	IIB	
270	60	80	HGSOC	1	*TP53*	c.326T>C	p.(Phe109Ser)	51	IIB	
271	53	50	HGSOC	1	*BRCA1*	c.3520_3821del	p.(Ser1174AsnfsTer12)	10	IA	
			HGSOC	1	*TP53*	c.659A>G	p.(Tyr220Cys)	11	IIB	
273	74	70	HGSOC	1	*TP53*	c.902del	p.(Pro301GlnfsTer44)	85	IIB	
276	58	70	HGSOC	2	*TP53*	c.848G>C	p.(Arg283Pro)	49	IIB	
278	71	80	HGSOC	1	*TP53*	c.824G>T	p.(Cys275Phe)	79	IIB	
281	66	20	HGSOC	1	*TP53*	c.814G>A	p.(Val272Met)	63	IIB	
			HGSOC	1	*TP53*	c.497C>G	p.(Ser166Ter)	17	IIB	
285	77	70	HGSOC	1	*TP53*	c.526del	p.(Cys176AlafsTer71)	58	IIB	
			HGSOC	1	*RB1*	c.1399C>T	p.(Arg467Ter)	33	X	
288	75	10	HGSOC	1	*BRCA2*	c.6275_6276del	p.(Leu2092ProfsTer7)	51	IA	x
291	82	50	MLAOC	1	*KRAS*	c.35G>A	p.(Gly12Asp)	46	IIB	
292	73	30	MOC	1	*KRAS*	c.35G>A	p.(Gly12Asp)	4	IIB	
			MOC	1	*PIK3CA*	c.3140A>G	p.(His1047Arg)	5	IIB	
293	60	60	HGSOC	1	*TP53*	c.766A>C	p.(Thr256Pro)	67	IIB	
294	77	40	HGSOC	1+2	*TP53*	c.730G>A	p.(Gly244Ser)	62	IIB	
			HGSOC	1+2	*RNF43*	c.60_94del	p.(Leu21GlyfsTer7)	27	X	
295	79	30	HGSOC	1	*TP53*	c.544del	p.(Cys182AlafsTer65)	57	IIB	
297	55	90	HGSOC	2	*BRCA1*	c.2118del	p.(Gly707ValfsTer29)	81	IA	
			HGSOC	2	*TP53*	c.1025G>C	p.(Arg342Pro)	68	IIB	

298	50	50	EOC	2	*BRCA1*	c.2507_2508del	p.(Glu836GlyfsTer2)	67	IA	
			EOC	2	*TP53*	c.533A>C	p.(His178Pro)	22	IIB	
			EOC	2	*PTEN*	c.407G>A	p.(Cys136Tyr)	23	IIB	
299	75	35	LGSOC	1	*TP53*	c.818G>A	p.(Arg273His)	50	IIB	
301	75	50	HGSOC	1	*TP53*	c.701_702delinsGA	p.(Tyr234Ter)	59	IIB	
304	66	20	HGSOC	1	*TP53*	c.659A>G	p.(Tyr220Cys)	29	IIB	
			HGSOC	1	*PIK3R1*	c.1690A>G	p.(Asn564Asp)	46	IIB	
306	74	75	HGSOC	2	*TP53*	c.722C>T	p.(Ser241Phe)	82	IIB	
			HGSOC	2	*BRCA1*	c.212G>T	p.(Arg71Met)	66	IA	
			HGSOC	2	*MET*	amp			IIIA	
307	53	60	HGSOC	1	*TP53*	c.853G>A	p.(Glu285Lys)	56	IIB	
			HGSOC	1	*RB1*	c.1128-1G>C	p.(?)	53	X	
312	83	90	HGSOC	1	*TP53*	c.818G>A	p.(Arg273His)	92	IIB	
319	74	20	HGSOC	1	*TP53*	c.923_930del	p.(Leu308GlnfsTer26)	6	IIB	
320	58	60	CCOC	1	*POLE*	c.857C>G	p.(Pro286Arg)	8	X	
321	69	30	HGSOC	2	*TP53*	c.818G>A	p.(Arg273His)	15	IIB	
323	43	15	HGSOC	1	*BRCA1*	c.3481_3491del	p.(Glu1161PhefsTer3)	66	IA	x
			HGSOC	1	*TP53*	c.919+1G>T	p.(?)	22	IIB	
325	64	70	CCOC	2	*PIK3CA*	c.1634A>C	p.(Glu545Ala)	30	IIB	
331	70	50	HGSOC	1	*TP53*	c.472_477del	p.(Arg158_Ala159del)	38	IIB	
334	64	40	HGSOC	1	*TP53*	c.517G>A	p.(Val173Met)	20	IIB	
			HGSOC	1	*TP53*	c.402T>A	p.(Phe134Leu)	12	IIB	
337	61	30	HGSOC	1	*TP53*	c.524G>A	p.(Arg175His)	50	IIB	
			HGSOC	1	*RB1*	c.2211+1G>A	p.(?)	56	X	
338	66	75	HGSOC	1	*TP53*	c.267del	p.(Ser90ProfsTer33)	53	IIB	
339	70	70	CCOC	2	*TP53*	c.659A>G	p.(Tyr220Cys)	82	IIB	
			CCOC	2	*PIK3CA*	c.1258T>C	p.(Cys420Arg)	27	IIB	
340	77	100	HGSOC	2	*TP53*	c.524G>A	p.(Arg175His)	81	IIB	
341	69	60	CCOC	2	*ATM*	c.7886_7890del	p.(Ile2629SerfsTer25)	40	IIB	
			CCOC	2	*CCND1*	amp			IVA	
343	67	50	HGSOC	1	*BRCA2*	c.8021dup	p.(Ile2675AspfsTer6)	5	IA	
			HGSOC	1	*TP53*	c.659A>G	p.(Tyr220Cys)	4	IIB	
344	63	10	HGSOC	2	*TP53*	c.483del	p.(Ile162SerfsTer8)	56	IIB	

348	57	50	HGSOC	1	*BRCA1*	c.4658T>A	p.(Leu1553Ter)	60	IA	
			HGSOC	1	*TP53*	c.833C>G	p.(Pro278Arg)	80	IIB	
349	82	20	HGSOC	2	*TP53*	c.725G>T	p.(Cys242Phe)	14	IIB	
352	60	30	HGSOC	1	*TP53*	c.659A>G	p.(Tyr220Cys)	42	IIB	
354	72	60	HGSOC	1	*ERBB2*	amp			IIB	
			HGSOC	1	*TP53*	c.723del	p.(Cys242AlafsTer5)	37	IIB	
356	67	90	HGSOC	1	*TP53*	c.422G>A	p.(Cys141Tyr)	80	IIB	
			HGSOC	1	*ERBB2*	amp			IIB	
			HGSOC	1	*AKT1*	c.145G>A	p.(Glu49Lys)	13	IIB	
357	72	50	HGSOC	1	*TP53*	c.505_534dup	p.(Met169_His178dup)	38	IIB	
			HGSOC	1	*PIK3R1*	c.1384_1385del	p.(Glu462IlefsTer2)	20	IIB	
			HGSOC	1	*PIK3R1*	c.1958_1959del	p.(Lys653ThrfsTer33)	10	IIB	
359	61	50	CCOC	2	*KRAS*	c.183A>C	p.(Gln61HIS))	30	IIB	
			CCOC	2	*ATM*	c.2098C>T	(p.Cys1626*)))	65	IIB	

in this cancer type   in other cancer types   in this cancer type and other cancer types   no evidence

Supplementary Table 3– overview of all genes included in the panels and matched drugs, clinical evidence and ESCAT ranking

Relevant therapy summary – *TP53* mutation (p53)							
**Relevant therapy**	**OncoKB**	**FDA**	**NCCN**	**EMA**	**Clinical trials**	**Pre-clinical**	**ESCAT score**
Atezolizumab and Bevacizumab (HGSOC)	⊘	⊘	⊘	⊘	●	●	II-B
APR-246 (HGSOC)	⊘	⊘	⊘	⊘	●	●	II-B
ZN-c3 and Bevacizumab ± Pembrolizumab	⊘	⊘	⊘	⊘	●	●	II-B
Adavosertib	⊘	⊘	⊘	⊘	●	●	II-B
CYH33 + Olaparib	⊘	⊘	⊘	⊘	●	●	II-B
PC14586 (+pembrolizumab) (Y220C only)	● [3]	⊘	⊘	⊘	●	●	II-B

Clinical Trial Summary – *TP53* mutation (p53)			
**NCT ID**	**Subject**	**phase**	**Results**
**NCT04510584**	Interventional Halting Early Advancement of Residual Disease With **Bevacizumab** and **Atezolizumab** as a maintenance treatment for patients with **TP53 mutant ovarian**, fallopian tube, or primary peritoneal cancer	II	2024
**NCT03268382**	PiSARRO-R: p53 Suppressor Activation in Platinum-Resistant High Grade **Serous Ovarian** Cancer, a Phase II Study of Systemic **Pegylated Liposomal Doxorubicin** Chemotherapy With APR-246	II	2022
**NCT05431582**	Phase I Study of **ZN-c3** and **Bevacizumab** ± **Pembrolizumab** in Metastatic CCNE1 Amplified and **TP53** mutant solid tumors	Ib	2025
**NCT01164995**	Phase II Pharmacological Study With **Wee-1 Inhibitor MK-1775** Combined With Carboplatin in Patients With **p53 Mutated** Epithelial Ovarian Cancer and Early Relapse (< 3 Months) or Progression During Standard First Line Treatment [1,2]	II	2022
**NCT04586335**	Open Label, Phase Ib Study to Evaluate the Safety, Tolerability, Pharmacokinetics and Clinical Activity **of CYH33**, an Oral PI3K Inhibitor in Combination With **Olaparib**, an Oral PARP Inhibitor in Patients With Advanced Solid Tumors	Ib	2024
**NCT04585750**	**PYNNACLE:** A Phase 1/2 Open-label, Multicenter Study to Assess the Safety, Tolerability, Pharmacokinetics, Pharmacodynamics, and Efficacy of **PC14586** in Patients With Advanced Solid Tumors Harboring **a p53 Y220C** Mutation (PYNNACLE)	I/II	2026

Pre - clinical Research Summary – *TP53* mutation (p53)		
**Title**	**model**	**journal**
Strong synergy with APR-246 and DNA-damaging drugs in primary cancer cells from patients with TP53 mutant High-Grade Serous ovarian cancer [3]	*in vitro*	Journal of Ovarian Research

🟢 in this cancer type 🟠 in other cancer types 🔵 in this cancer type and other cancer types ⚪ no evidence

Relevant therapy summary – *BCRA1/2* mutation (HR)							
**Relevant therapy**	**OncoKB**	**FDA**	**NCCN**	**EMA**	**Clinical trials**	**Pre-clinical**	**ESCAT score**
Olaparib	🔵 1	🔵	🔵	🔵	🔵	🔵	I-A
Rucaparib	🔵 1	🔵	🔵	⚪	🔵	🔵	I-A
Niraparib	🔵 1	🟢	🟢	🟢	🔵	🔵	I-A
Talazoparib	🟠 3	🟠	🟠	🟠	🔵	🔵	III-A
Olaparib + Cediranib	⚪	⚪	⚪	⚪	🔵	🔵	V
Olaparib + Ceralasertib	⚪	⚪	⚪	⚪	🔵	🔵	V
Niraparib + Atezolizumab	⚪	⚪	⚪	⚪	🔵	🔵	V
Niraparib + Dostarlimab	⚪	⚪	⚪	⚪	🔵	🔵	V
CYH33 + Olaparib	⚪	⚪	⚪	⚪	🔵	🔵	V

Clinical Trial Summary – *BRCA1/2* mutation (HR)			
**NCT ID**	**Subject**	**phase**	**Results**
**NCT03598270**	**ANITA**: A Phase III **Randomized**, Double-blinded Trial of Platinum-based Chemotherapy With or Without **Atezolizumab** Followed by **Niraparib** Maintenance With or Without Atezolizumab in Patients With Recurrent **Ovarian**, Tubal or Peritoneal Cancer and Platinum Treatment-free Interval (TFIp) >6 Months [4]	III	2023
**NCT03278717**	**ICON9**: International Phase III **Randomized** Study to Evaluate the Efficacy of Maintenance Therapy With Olaparib and **Cediranib** or Olaparib Alone in Patients With Relapsed Ovarian Cancer Following a Response to Platinum-based Chemotherapy [5]	III	2023
**NCT03462342**	**CAPRI**: interventional **non-randomized** phase 2 trial to evaluate safety and tolerability and also to determine the ORR and PFS of combination of **AZD6738** (ceralasertib) and **Olaparib** in women with recurrent ovarian cancer in distinct platinum-sensitive and platinum-resistant cohorts	II	2022
**NCT02477644**	**PAOLA-1**: **Randomized**, Double-Blind, Phase III Trial of **Olaparib** vs. Placebo in Patients with Advanced FIGO Stage IIIB - IV High Grade Serous or Endometrioid Ovarian, Fallopian Tube, or Peritoneal Cancer treated with standard First-Line Treatment, Combining Platinum-Taxane Chemotherapy and **Bevacizumab** Concurrent with Chemotherapy and in Maintenance [6,7]	III	2022
**NCT02655016**	**PRIMA**: A Phase 3, Randomized, Double-Blind, Placebo-Controlled, Multicenter Study of Niraparib Maintenance Treatment in Patients With Advanced Ovarian Cancer Following Response on Front-Line Platinum-Based Chemotherapy [8–11]	III	2022
**NCT01844986**	**SOLO-1**: A Phase III, **Randomized,** Double Blind, Placebo Controlled, Multicentre Study of **olaparib** Maintenance Monotherapy in Patients With **BRCA Mutated** Advanced (FIGO Stage III-IV) Ovarian Cancer Following First Line Platinum Based Chemotherapy [12–15]	III	2019
**NCT04586335**	Open Label, Phase Ib Study to Evaluate the Safety, Tolerability, Pharmacokinetics and Clinical Activity of **CYH33**, an Oral PI3K Inhibitor in Combination With **Olaparib**, an Oral PARP Inhibitor in Patients With Advanced Solid Tumors	Ib	2024
NCT02925234	DRUP: This is a prospective, non-randomized clinical trial that aims to describe the efficacy and toxicity of commercially available, **targeted anticancer drugs** prescribed for treatment of patients with **advanced cancer** with a **potentially actionable** variant as revealed by a genomic or protein expression test.	II	2027
NCT02693535	**TAPUR: Testing** the Use of Food and Drug Administration (FDA) **Approved Drugs** That **Target** a Specific **Abnormality** in a **Tumor Gene** in People With Advanced Stage **Cancer (TAPUR)**	II	2025

Pre - clinical Research Summary – *BRCA1/2* mutation (HR)		
Title	**model**	**journal**
Ceralasertib-Mediated ATR Inhibition Combined With Olaparib in Advanced Cancers Harboring DNA Damage Response and Repair Alterations (Olaparib Combinations) [16]	in patiënts	JCO Precision Oncology
ATR Inhibitor AZD6738 (Ceralasertib) Exerts Antitumor Activity as a Monotherapy and in Combination with Chemotherapy and the PARP Inhibitor Olaparib [17]	in vitro + in vivo	Cancer Research

green: in this cancer type | orange: in other cancer types | blue: in this cancer type and other cancer types | grey: no evidence

Relevant therapy summary – *ATM* mutation (HR)							
**Relevant therapy**	**OncoKb**	**FDA**	**NCCN**	**EMA**	**Clinical trials**	**Pre-clinical**	**ESCAT score**
Olaparib	orange [1]	orange	orange	grey	blue	blue	III-A
talazoparib	orange [1]	orange	orange	grey	blue	blue	III-A
Rucaparib + Atezolizumab	grey	grey	grey	grey	blue	blue	V
Elimusertib + Pembrolizumab	grey	grey	grey	grey	blue	blue	V
Elimusertib + Niraparib	grey	grey	grey	grey	blue	blue	V
Rucaparib + Nivolumab	grey	grey	grey	grey	green	blue	V
pembrolizumab + Olaparib	grey	grey	grey	grey	orange	blue	V
Ipilimumab + Nivolumab	grey	orange	grey	grey	blue	blue	III-A

Clinical Trial Summary – *ATM* mutation (HR)			
**NCT ID**	**Subject**	**phase**	**Results**
NCT04276376	**ARIANES**: A Multicenter, Open Label, Phase II Basket Trial Exploring the Efficacy And Safety of The Combination of **Rucaparib** (PARP Inhibitor) And **Atezolizumab** (Anti-PD-L1 Antibody) In Patients With **DNA Repair- Deficient** or Platinum-Sensitive Solid Tumors	II	2025
NCT04095273	A Multicenter, **Non-randomized**, Open-label Phase Ib Study to Determine the Maximum Tolerated and Recommended Phase II Dose of the ATR Inhibitor **BAY1895344** in Combination With **Pembrolizumab** and to Characterize Its Safety, Tolerability, Pharmacokinetics and **Preliminary Anti-tumor Activity** in Patients With **Advanced Solid Tumors**	Ib	2023
NCT04267939	An Open-label Phase Ib Study to Determine the Maximum Tolerated and/or Recommended Phase II Dose of the **ATR Inhibitor BAY 1895344** in Combination With PARP Inhibitor **Niraparib**, in Patients With Recurrent Advanced Solid Tumors and **Ovarian Cancer** [18]	Ib	2025
NCT03522246	**ATHENA**: This is a Phase 3, **randomized**, multinational, double-blind, dual placebo-controlled, 4-arm study evaluating **rucaparib** and **nivolumab** as maintenance treatment following response to front-line treatment in newly diagnosed **ovarian cancer** patients. Response to treatment will be analyzed based on **homologous recombination (HR) status** of tumor samples.	III	2030
NCT03207347	Non-randomized interventional Phase II trial of the **PARP inhibitor**, niraparib, in **BAP1** and other DNA damage response **(DDR)** pathway deficient neoplasms [19]	II	2023
NCT04666740	**POLAR**: A non-randomized Phase 2 Trial to Evaluate the Safety and Antitumor Activity of **Pembrolizumab** and **Olaparib** (**POLAR**) Maintenance for Patients With Metastatic Pancreatic Ductal Adenocarcinoma and Homologous Recombination Deficiency and/or Exceptional Treatment Response to Platinum-Based Therapy [20]	II	2024
NCT02925234	**DRUP**: This is a prospective, non-randomized clinical trial that aims to describe the efficacy and toxicity of commercially available, **targeted anticancer drugs** prescribed for treatment of patients with **advanced cancer** with a **potentially actionable** variant as revealed by a genomic or protein expression test.	II	2027
NCT02693535	**TAPUR: Testing** the Use of Food and Drug Administration (FDA) **Approved Drugs** That **Target** a Specific **Abnormality** in a **Tumor Gene** in People With Advanced Stage **Cancer (TAPUR)**	II	2025

Pre - clinical Research Summary – *ATM* mutation (HR)		
Title	**model**	**journal**
Damage Incorporated: Discovery of the Potent, Highly Selective, Orally Available ATR Inhibitor BAY 1895344 with Favorable Pharmacokinetic Properties and Promising Efficacy in Monotherapy and in Combination Treatments in Preclinical Tumor Models [21]	*in vivo*	Journal of Medicinal Chemistry
First-in-Human Trial of the Oral Ataxia Telangiectasia and RAD3-Related (ATR) Inhibitor BAY 1895344 in Patients with Advanced Solid Tumors [22]	*in vivo + in patients*	Cancer Discovery

Relevant therapy summary – *BAP1* mutation (HR)							
**Relevant therapy**	**OncoKb**	**FDA**	**NCCN**	**EMA**	**Clinical trials**	**Pre-clinical**	**ESCAT score**
Niraparib	grey	grey	grey	grey	orange	blue	III-B
Olaparib + Pembrolizumab	grey	grey	grey	grey	orange	blue	III-B
Rucaparib	grey	grey	grey	grey	orange	blue	III-B

(green) in this cancer type (orange) in other cancer types (blue) in this cancer type and other cancer types (grey) no evidence

Clinical Trial Summary – *BAP1* mutation (HR)

NCT ID	Subject	phase	Results
NCT03207347	**UF-STO-ETI-001**: Non-randomized interventional Phase II trial of the PARP inhibitor, **niraparib,** in **BAP1** and other DNA damage response (**DDR**) pathway deficient neoplasms [19]	II	2023
NCT04666740	**POLAR**: A non-randomized Phase 2 Trial to Evaluate the Safety and Antitumor Activity of Pembrolizumab and OLApaRib (POLAR) Maintenance for Patients With Metastatic Pancreatic Ductal Adenocarcinoma and Homologous Recombination Deficiency and/or Exceptional Treatment Response to Platinum-Based Therapy [20]	II	2024
NCT03654833	**MIST**: A Stratified Multi-arm Phase IIa Clinical Trial to Enable Accelerated Evaluation of Targeted Therapies for Relapsed Malignant Mesothelioma [23]	IIa	2023
NCT02925234	DRUP: This is a prospective, non-randomized clinical trial that aims to describe the efficacy and toxicity of commercially available, **targeted anticancer drugs** prescribed for treatment of patients with **advanced cancer** with a **potentially actionable** variant as revealed by a genomic or protein expression test.	II	2027

Pre - clinical Research Summary – *BAP1* mutation (HR)

Title	model	journal
Pyruvate dehydrogenase inactivation causes glycolytic phenotype in BAP1 mutant uveal melanoma [24]	in vitro	Oncogene
First-in-Human Trial of the Oral Ataxia Telangiectasia and RAD3-Related (ATR) Inhibitor BAY 1895344 in Patients with Advanced Solid Tumors [22]	in vivo + in patients	Cancer Discovery
BAP1-Altered Malignant Pleural Mesothelioma: Outcomes With Chemotherapy, Immune Check-Point Inhibitors and Poly(ADP-Ribose) Polymerase Inhibitors [25]	In patients	Frontiers in Oncology

Relevant therapy summary – *ARID1A* mutation (HR)

Relevant therapy	OncoKB	FDA	NCCN	EMA	Clinical trials	Pre-clinical	ESCAT score
Niraparib	grey	blue	blue	grey	blue	blue	I-C
PLX2853 (+ Carboplatin)	blue [4]	grey	grey	grey	blue	blue	II-B
Tazemetostat (CCOC)	blue [4]	orange	orange	grey	blue	blue	II-B
Niraparib + Bevacizumab	grey	grey	grey	grey	blue	blue	II-B
IR + Olaparib	grey	grey	grey	grey	blue	blue	II-B
AZD6738 + Olaparib	grey	grey	grey	grey	blue	blue	II-B
Nivolumab	grey	grey	grey	grey	blue	blue	II-B
CPI-0209	grey	grey	grey	grey	blue	blue	II-B

(green) in this cancer type (orange) in other cancer types (blue) in this cancer type and other cancer types (grey) no evidence

Clinical Trial Summary – *ARID1A* mutation (HR)			
**NCT ID**	**Subject**	**phase**	**Results**
NCT03207347	Non-randomized interventional Phase II trial of the PARP inhibitor, niraparib, in BAP1 and other DNA damage response (DDR) pathway deficient neoplasms [19]	II	2023
NCT05523440	Interventional randomized phase 2 trial of niraparib or niraparib and bevacizumab combination in patients with recurrent endometrial cancer and/or ovarian cancer with ARID1A mutation (OU-SCC-ARID1A)	II	2026
NCT04493619	A Multicenter, Open-Label, Parallel, Phase 2a Study of PLX2853 Monotherapy in Advanced Gynecological Malignancies With a Known ARID1A Mutation and Phase 1b/2a Study of PLX2853/Carboplatin Combination Therapy in Platinum-Resistant Epithelial Ovarian Cancer [26]	IIa	2022
NCT03348631	interventional Phase II Study of Tazemetostat (EPZ-6438) (IND # 138671) in Recurrent or Persistent Endometrioid or Clear Cell Carcinoma of the Ovary, and Recurrent or Persistent Endometrioid Endometrial Adenocarcinoma [27]	II	2025
NCT05023655	A Phase II Study of Tazemetostat in Solid Tumors Harboring an ARID1A Mutation 2829303132	II	2025
NCT03682289	Interventional non-randomized Phase II Trial of AZD6738 Alone and in Combination With Olaparib in Patients With Selected Solid Tumor Malignancies [33]	II	2023
NCT04065269	ATARI: interventional non-randomized trial with ATR Inhibitor in Combination With Olaparib in Gynecological Cancers With ARID1A Loss [34]	II	2023
NCT04957615	A Phase II Clinical Trial to Investigate ARID1A Mutation and CXCL13 Expression in the Pre-Treatment Tumor Samples as a Combinatorial Predictive Biomarker for Immune Checkpoint Therapy in Metastatic Solid Tumors[33]	II	2023
NCT04104776	A Phase 1/2 Study of CPI-0209 in Patients With Advanced Solid Tumors and Lymphomas [33,35]	II	2026

Pre - clinical Research Summary – *ARID1A* mutation (HR)		
**Title**	**model**	**journal**
Treating ARID1A mutated cancers by harnessing synthetic lethality and DNA damage response [26]	*in vitro*	Oncogene
Loss of ARID1A in Tumor Cells Renders Selective Vulnerability to Combined Ionizing Radiation and PARP Inhibitor Therapy [36]	*in vivo+ in vitro*	Clinical cancer research
Treatment Strategies for ARID1A-Deficient Ovarian Clear Cell Carcinoma [27]	*In silico*	Cancers
Selective sensitivity of EZH2 inhibitors based on synthetic lethality in ARID1A-deficient gastric cancer[30]	*In vitro*	Gastric cancer
ARID1A determines luminal identity and therapeutic response in estrogen-receptor-positive breast cancer[32]	*In silico*	Nature Genetics
Synthetic lethality by targeting EZH2 methyltransferase activity in ARID1A-mutated cancers[31]	*In vitro*	Nature Medicine
ARID1A Mutation in Metastatic Breast Cancer: A Potential Therapeutic Target [33]	*In silico*	Frontiers in Oncology
ATR Inhibitor AZD6738 (Ceralasertib) Exerts Antitumor Activity as a Monotherapy and in Combination with Chemotherapy and the PARP Inhibitor Olaparib [17]	*In vivo*	Cancer research

Relevant therapy summary – *AKT1* mutation (PI3/AKT/mTOR)							
**Relevant therapy**	**OncoKB**	**FDA**	**NCCN**	**EMA**	**Clinical trials**	**Pre-clinical**	**ESCAT score**
Capivasertib (E17K) (AZD5363)	blue [3]	grey	grey	grey	blue	green	II-B
temsirolimus	grey	grey	grey	grey	blue	green	II-B
Alpelisib	grey	grey	grey	grey	blue	grey	II-B
capivasertib + fulvestrant (E17K)	grey	grey	grey	grey	orange	green	II-B
ipatasertib* + paclitaxel	grey	grey	grey	orange	blue	green	II-B
ipatasertib* + atezolizumab	grey	grey	grey	grey	blue	green	II-B

*specific pediatric waiver for breast and prostate cancer

(green) in this cancer type (orange) in other cancer types (blue) in this cancer type and other cancer types (grey) no evidence

Clinical Trial Summary – *AKT1* mutation (PI3/AKT/mTOR)

NCT ID	Subject	phase	Results
NCT02523014	Phase II Trial of SMO/ AKT/ NF2/CDK Inhibitors in Progressive Meningiomas With SMO/ AKT/ NF2/CDK Pathway Mutations	II	2024
NCT01226316	A Phase I, Open-Label, Multicenter Study to Assess the Safety, Tolerability, Pharmacokinetics and Preliminary Anti-tumor Activity of Ascending Doses of AZD5363 Under Adaptable Dosing Schedules in Patients With Advanced Solid Malignancies	I	2023
NCT04931342	**BOUQUET:** A Phase II, Open-Label, Multicenter, Platform Study Evaluating the Efficacy and Safety of Biomarker-Driven Therapies in Patients With Persistent or Recurrent Rare Epithelial Ovarian Tumors	II	2026
NCT04632992	**MyTACTIC:** A Study Evaluating Targeted Therapies in Participants Who Have Advanced Solid Tumors With Genomic Alterations or Protein Expression Patterns Predictive of Response	II	2024
NCT02465060	**MATCH:** phase II trial to evaluate the proportion of patients with objective response (OR) to targeted study agent(s) in patients with advanced refractory cancers/lymphomas/multiple myeloma	II	2025
NCT02693535	**TAPUR: Testing** the Use of Food and Drug Administration (FDA) **Approved Drugs** That **Target** a Specific **Abnormality** in a **Tumor Gene** in People With Advanced Stage **Cancer (TAPUR)**	II	2025
NCT02925234	DRUP: This is a prospective, non-randomized clinical trial that aims to describe the efficacy and toxicity of commercially available, **targeted anticancer drugs** prescribed for treatment of patients with **advanced cancer** with a **potentially actionable** variant as revealed by a genomic or protein expression test.	II	2027

Pre - clinical Research Summary – *AKT1* mutation (HR)

Title	model	journal
A Phase I Open-Label Study to Identify a Dosing Regimen of the Pan-AKT Inhibitor AZD5363 for Evaluation in Solid Tumors and in PIK3CA-Mutated Breast and Gynecologic Cancers [37]	*In patient*	Clinical cancer research
Capivasertib, an AKT Kinase Inhibitor, as Monotherapy or in Combination with Fulvestrant in Patients with AKT1 E17K-Mutant, ER-Positive Metastatic Breast Cancer [38]	*In patient*	Clinical cancer research
Comparing PI3K/Akt Inhibitors Used in Ovarian Cancer Treatment [39]	*In vitro*	Frontiers In Pharmacology

Relevant therapy summary – *PIK3CA* mutation (PI3/AKT/mTOR)

Relevant therapy	OncoKB	FDA	NCCN	EMA	Clinical trials	Pre-clinical	ESCAT score
Alpelisib	(grey)	(orange)	(orange)	(orange)	(blue)	(blue)	III-A
Alpelisib + Fulvestrant (E542K, E545A, E545D, E545G, E545K, H104L, H1047R, H1047Y, Q546E, Q546R, C420R)	(orange) 1	(orange)	(orange)	(orange)	(blue)	(blue)	II-B
Capivasertib*	(grey)	(grey)	(grey)	(orange)	(blue)	(blue)	II-B
TQ-B3525	(grey)	(grey)	(grey)	(grey)	(blue)	(grey)	III-A
temsirolimus	(grey)	(grey)	(grey)	(grey)	(blue)	(grey)	III-A
Copanlisib + Fulvestrant	(grey)	(orange)	(orange)	(grey)	(blue)	(orange)	III-A
CYH33 (+ Olaparib) (E542K, 1047R or E545K)	(grey)	(grey)	(grey)	(grey)	(blue)	(orange)	II-IA
Ipatasertib + Paclitaxel	(grey)	(grey)	(grey)	(grey)	(blue)	(orange)	II-B
Inavolisib + Palbociclib (+ Letrozole)	(grey)	(grey)	(grey)	(grey)	(blue)	(grey)	II-B

*specific pediatric waiver for breast and prostate cancer

● in this cancer type ● in other cancer types ● in this cancer type and other cancer types ⊘ no evidence

Clinical Trial Summary – *PIK3CA* mutation (PI3/AKT/mTOR)

NCT ID	Subject	phase	Results
NCT01226316	**Phase I**, Open-Label, Multicenter Study to Assess the Safety, Tolerability, Pharmacokinetics and Preliminary Anti-tumor Activity of Ascending Doses of **AZD5363** Under Adaptable Dosing Schedules in Patients With Advanced Solid Malignancies [37]	I	2018
NCT02437318	**SOLAR-1**: **phase 3**, randomized, double-blind, placebo-controlled trial of **Alpelisib** plus **fulvestrant** versus placebo plus Fulvestrant in 572 patients including postmenopausal women, and men, with HR-positive, **HER2-negative**, advanced or metastatic **breast** cancer	III	2023
NCT04836663	Open-label, Multicenter **Phase II** Study of **TQ-B3525** Tablets in Subjects With **PIK3CA** and/or **PIK3R1/2** Gene-altered Recurrent/Metastatic Advanced **Gynecologic Tumors**	II	2023
NCT05082025	**Phase 2** Study of PI3K Inhibitor **Copanlisib** in Combination With Fulvestrant in Selected ER+ and/or PR+ Cancers With PI3K **(PIK3CA, PIK3R1)** and/or **PTEN** Alterations	II	2026
NCT05043922	**Phase II,** Open-Label, Multicenter Study to Evaluate the Efficacy and Safety of **CYH33**, a Selective PI3Kα Inhibitor in Patients With Recurrent/Persistent **Ovary**, Fallopian Tube or Primary Peritoneal **Clear Cell Carcinoma**	II	2023
NCT04586335	Open Label, **Phase Ib** Study to Evaluate the Safety, Tolerability, Pharmacokinetics and Clinical Activity of **CYH33**, an Oral PI3K Inhibitor in Combination With **Olaparib**, an Oral PARP Inhibitor in Patients With Advanced Solid Tumors	Ib	2024
NCT04931342	**BOUQUET: Phase II**, Open-Label, Multicenter, Platform Study Evaluating the Efficacy and Safety of Biomarker-Driven Therapies in Patients With Persistent or Recurrent **Rare Epithelial Ovarian Tumors**	II	2026
NCT04632992	**MyTACTIC**: A Study Evaluating Targeted Therapies in Participants Who Have Advanced Solid Tumors With Genomic Alterations or Protein Expression Patterns Predictive of Response	II	2024
NCT02925234	**DRUP**: This is a prospective, non-randomized clinical trial that aims to describe the efficacy and toxicity of commercially available, **targeted anticancer drugs** prescribed for treatment of patients with **advanced cancer** with a **potentially actionable** variant as revealed by a genomic or protein expression test.	II	2027
NCT02693535	**TAPUR: Testing** the Use of Food and Drug Administration (FDA) **Approved Drugs** That **Target** a Specific **Abnormality** in a **Tumor Gene** in People With Advanced Stage **Cancer (TAPUR)**	II	2025

Pre - clinical Research Summary – *PIK3CA* mutation (PI3/AKT/mTOR)

Title	model	journal
EPIK-O/ENGOT-OV61: Alpelisib plus Olaparib vs cytotoxic chemotherapy in high-grade serous ovarian cancer (phase III study) [40]	In silico<br>In patient	Future oncology
Development of PI3K inhibitors: Advances in clinical trials and new strategies (Review) [41]	Summary	Pharmacological Research
Spotlight on Copanlisib and its potential in the treatment of relapsed/refractory follicular lymphoma: Evidence to date [42]	In vivo<br>In patient	Onco Targets
Ipatasertib plus paclitaxel for *PIK3CA/AKT1/PTEN*-altered hormone receptor-positive *HER2*-negative advanced breast cancer: primary results from cohort B of the IPATunity130 randomized phase 3 trial [43]	In patient	Breast cancer treatment and research

Relevant therapy summary – *PIK3CA* amplification (PI3/AKT/mTOR)

Relevant therapy	OncoKB	FDA	NCCN	EMA	Clinical trials	Pre-clinical	ESCAT score
TQ-B3525	⊘	⊘	⊘	⊘	●	⊘	III-B
Copanlisib	⊘	⊘	⊘	⊘	●	⊘	III-B
Everolimus	⊘	⊘	⊘	⊘	●	⊘	V

Clinical Trial Summary – *PIK3CA* amplification (PI3/AKT/mTOR)

NCT ID	Subject	phase	Results
NCT04836663	Open-label, Multicenter Phase II Study of TQ-B3525 Tablets in Subjects With *PIK3CA* and/or *PIK3R1/2* Gene-altered Recurrent/Metastatic Advanced Gynecologic Tumors	II	2023
NCT05082025	Phase 2 Study of PI3K Inhibitor Copanlisib in Combination With Fulvestrant in Selected ER+ and/or PR+ Cancers With PI3K (*PIK3CA, PIK3R1*) and/or *PTEN* Alterations	II	2026
NCT02449538	a single-arm, open-label phase II trial of Everolimus in *PIK3CA* amplification/mutation and/or **PTEN** loss patients with advanced solid tumors refractory to standard therapy	II	2017

🟢 in this cancer type 🟠 in other cancer types 🔵 in this cancer type and other cancer types ⊘ no evidence

Relevant therapy summary – *PIK3R1* mutation (PI3/AKT/mTOR)

Relevant therapy	OncoKB	FDA	NCCN	EMA	Clinical trials	Pre-clinical	ESCAT score
TQ-B3525	⊘	⊘	⊘	⊘	🔵	⊘	II-B
Copanlisib	⊘	⊘	⊘	⊘	🔵	⊘	II-B
Alpelisib	⊘	🟠	🟠	🟠	🔵	🔵	III-A

Clinical Trial Summary – *PIK3R1* mutation (PI3/AKT/mTOR)

NCT ID	Subject	phase	Results
NCT04836663	Open-label, Multicenter Phase II Study of TQ-B3525 Tablets in Subjects With *PIK3CA* and/or *PIK3R1/2* Gene-altered Recurrent/Metastatic Advanced Gynecologic Tumors	II	2022
NCT05082025	Phase 2 Study of PI3K Inhibitor **Copanlisib** in Combination With Fulvestrant in Selected ER+ and/or PR+ Cancers With PI3K **(PIK3CA, PIK3R1)** and/or **PTEN** Alterations	II	2026
NCT02925234	DRUP: This is a prospective, non-randomized clinical trial that aims to describe the efficacy and toxicity of commercially available, **targeted anticancer drugs** prescribed for treatment of patients with **advanced cancer** with a **potentially actionable** variant as revealed by a genomic or protein expression test.	II	2027

Relevant therapy summary – *PTEN* mutation (PI3/AKT/mTOR)

Relevant therapy	OncoKB	FDA	NCCN	EMA	Clinical trials	Pre-clinical	ESCAT score
Niraparib	⊘	⊘	⊘	⊘	🔵	⊘	II-B
GSK2636771, AZD8186	🔵[4]	⊘	⊘	⊘	🔵	⊘	II-B
Copanlisib	⊘	⊘	⊘	⊘	🔵	⊘	II-B
Alpelisib	⊘	🟠	🟠	🟠	🔵	🔵	IIIA
Ipatasertib (+ Paclitaxel)	⊘	⊘	⊘	⊘	🔵	⊘	II-B
Ipatasertib + Atezolizumab	⊘	⊘	⊘	⊘	🔵	⊘	II-B
temsirolimus	⊘	⊘	⊘	⊘	🔵	⊘	II-B
Everolimus	⊘	⊘	⊘	⊘	🔵	⊘	V

Clinical Trial Summary – *PTEN* mutation (PI3/AKT/mTOR)

NCT ID	Subject	phase	Results
NCT03207347	Non-randomised interventional Phase II trial of the PARP inhibitor, niraparib, in **BAP1** and other DNA damage response (DDR) pathway deficient neoplasms [19]	II	2023
NCT04931342	**BOUQUET: Phase II**, Open-Label, Multicenter, Platform Study Evaluating the Efficacy and Safety of Biomarker-Driven Therapies in Patients With Persistent or Recurrent **Rare Epithelial Ovarian Tumors**	II	2026
NCT04632992	**MyTACTIC**: A Study Evaluating Targeted Therapies in Participants Who Have Advanced Solid Tumors With Genomic Alterations or Protein Expression Patterns Predictive of Response	II	2024
NCT02693535	**TAPUR: Testing** the Use of Food and Drug Administration (FDA) **Approved Drugs** That **Target** a Specific **Abnormality** in a **Tumor Gene** in People With Advanced Stage **Cancer (TAPUR)**	II	2025
NCT05082025	**Phase 2** Study of PI3K Inhibitor **Copanlisib** in Combination With Fulvestrant in Selected ER+ and/or PR+ Cancers With PI3K **(PIK3CA, PIK3R1)** and/or **PTEN** Alterations	II	2026
NCT02449538	a single-arm, open-label phase II trial of everolimus in PIK3CA amplification/mutation and/or **PTEN** loss patients with advanced solid tumors refractory to standard therapy	II	2017
NCT02925234	DRUP: This is a prospective, non-randomized clinical trial that aims to describe the efficacy and toxicity of commercially available, **targeted anticancer drugs** prescribed for treatment of patients with **advanced cancer** with a **potentially actionable** variant as revealed by a genomic or protein expression test.	II	2027

in this cancer type in other cancer types in this cancer type and other cancer types no evidence

Relevant therapy summary – *ERBB2* mutation (ErbB kinases)							
**Relevant therapy**	**OncoKB**	**FDA**	**NCCN**	**EMA**	**Clinical trials**	**Pre-clinical**	**ESCAT score**
Trastuzumab (+ combinations)	[1]						II-B
Alpelisib + Fulvestrant							II-B
Afatinib							II-B
Neratinib							II-B

Clinical Trial Summary – *ERBB2* mutation (ErbB kinases)			
**NCT ID**	**Subject**	**phase**	**Results**
NCT02437318	SOLAR-1: a phase 3, randomized, double-blind, placebo-controlled trial of alpelisib plus fulvestrant versus placebo plus fulvestrant in 572 patients including postmenopausal women, and men, with HR-positive, HER2-negative, advanced or metastatic breast cancer	III	2023
NCT04931342	BOUQUET: A Phase II, Open-Label, Multicenter, Platform Study Evaluating the Efficacy and Safety of Biomarker-Driven Therapies in Patients With Persistent or Recurrent Rare Epithelial Ovarian Tumors	II	2026
NCT04632992	MyTACTIC: A Study Evaluating Targeted Therapies in Participants Who Have Advanced Solid Tumors With Genomic Alterations or Protein Expression Patterns Predictive of Response	II	2024
NCT02465060	MATCH: phpase II trial to evaluate the proportion of patients with objective response (OR) to targeted study agent(s) in patients with advanced refractory cancers/lymphomas/multiple myeloma	II	2025
NCT02693535	TAPUR: Testing the Use of Food and Drug Administration (FDA) Approved Drugs That Target a Specific Abnormality in a Tumor Gene in People With Advanced Stage Cancer (TAPUR)	II	2025
NCT02925234	DRUP: This is a prospective, non-randomized clinical trial that aims to describe the efficacy and toxicity of commercially available, targeted anticancer drugs prescribed for treatment of patients with advanced cancer with a potentially actionable variant as revealed by a genomic or protein expression test.	II	2027

Relevant therapy summary – *ERBB2* amplification (ErbB kinases)							
**Relevant therapy**	**OncoKB**	**FDA**	**NCCN**	**EMA**	**Clinical trials**	**Pre-clinical**	**ESCAT score**
Lapatinib + Capecitabine, Lapatinib + Letrozole							II-B
Margetuximab + Chemotherapy							II-B
Neratinib, Neratinib + Capecitabine							II-B
Trastuzumab (+ combinations)							II-B

green: in this cancer type — orange: in other cancer types — blue: in this cancer type and other cancer types — grey: no evidence

Clinical Trial Summary – *ERBB2* amplification (ErbB kinases)

NCT ID	Subject	phase	Results
NCT02437318	**SOLAR-1**: a phase 3, randomized, double-blind, placebo-controlled trial of alpelisib plus fulvestrant versus placebo plus fulvestrant in 572 patients including postmenopausal women, and men, with HR-positive, HER2-negative, advanced or metastatic breast cancer	III	2023
NCT04931342	**BOUQUET**: A Phase II, Open-Label, Multicenter, Platform Study Evaluating the Efficacy and Safety of Biomarker-Driven Therapies in Patients With Persistent or Recurrent Rare Epithelial Ovarian Tumors	II	2026
NCT04632992	**MyTACTIC**: A Study Evaluating Targeted Therapies in Participants Who Have Advanced Solid Tumors With Genomic Alterations or Protein Expression Patterns Predictive of Response	II	2024
NCT02693535	**TAPUR: Testing** the Use of Food and Drug Administration (FDA) **Approved Drugs** That **Target** a Specific **Abnormality** in a **Tumor Gene** in People With Advanced Stage **Cancer (TAPUR)**	II	2025
NCT02925234	**DRUP**: This is a prospective, non-randomized clinical trial that aims to describe the efficacy and toxicity of commercially available, **targeted anticancer drugs** prescribed for treatment of patients with **advanced cancer** with a **potentially actionable** variant as revealed by a genomic or protein expression test.	II	2027
NCT03219268	A Phase 1, First-in-Human, Open-Label, Dose Escalation Study of **MGD013**, A Bispecific DART® Protein Binding PD-1 and LAG-3 in Patients With Unresectable or Metastatic Neoplasms	I	2023
NCT04482309	**DESTINY-PanTumor02**: A Phase 2, Multicenter, Open-label Study to Evaluate the Efficacy and Safety of **Trastuzumab** Deruxtecan (T-DXd, DS-8201a) for the Treatment of Selected HER2 Expressing Tumors	II	2027

Relevant therapy summary – *KRAS* mutation (RTK/RAS/MAPK)

Relevant therapy	OncoKB	FDA	NCCN	EMA	Clinical trials	Pre-clinical	ESCAT score
Sotorasib (G12C)	orange 1	orange	orange	orange	orange	blue	III-A
Cobimetinib, Trametinib	orange 2	grey	grey	grey	orange	blue	III-A
Adagrasib (G12C)	orange 1	orange	orange	grey	orange	blue	III-A
Adagrasib, Adagrasib + Cetuximab (G12C)	orange 3	grey	grey	grey	orange	blue	III-A
Trametinib, Cobimetinib, Binimetinib	blue 4	grey	grey	grey	blue	blue	III-A
Cobimetinib	grey	grey	grey	grey	blue	blue	II-B
atezolizumab	grey	orange	grey	grey	orange	blue	II-B
Avutometinib (+ defactinib)	grey	grey	grey	grey	green	green	II-B

Clinical Trial Summary – *KRAS* mutation (RTK/RAS/MAPK)

NCT ID	Subject	phase	Results
NCT04931342	**BOUQUET**: A Phase II, Open-Label, Multicenter, Platform Study Evaluating the Efficacy and Safety of Biomarker-Driven Therapies in Patients With Persistent or Recurrent Rare Epithelial Ovarian Tumors	II	2026
NCT04625270	**RAMP-201:**A Phase 2 Study of Avutometinib (VS-6766) (Dual RAF/MEK Inhibitor) Alone and In Combination With Defactinib (FAK Inhibitor) in Recurrent Low-Grade Serous Ovarian Cancer (LGSOC)<br>**RAMP-301: announced July**	II<br>III	2025
NCT03875820	**FRAME**: A Phase I Trial of the Combination of Defactinib (VS-6063) (FAK Inhibitor) and VS-6766 (RO5126766) (CH5126776) (a Dual RAF/MEK Inhibitor) in Patients With Advanced Solid Tumours	I	2023

in this cancer type | in other cancer types | in this cancer type and other cancer types | no evidence

Relevant therapy summary – *NRAS* mutation (RTK/RAS/MAPK)							
**Relevant therapy**	**OncoKB**	**FDA**	**NCCN**	**EMA**	**Clinical trials**	**Pre-clinical**	**ESCAT score**
Cobimetinib, Trametinib	in other cancer types (3)	no evidence	no evidence	no evidence	in other cancer types	in this cancer type and other cancer types	III-A
trametinib	no evidence	no evidence	no evidence	no evidence	in this cancer type and other cancer types	in this cancer type and other cancer types	II-B
Binimetinib	in other cancer types (3)	no evidence	no evidence	no evidence	in this cancer type and other cancer types	in this cancer type and other cancer types	II-B
Selumetinib + Iodine I 131-6-Beta-Iodomethyl -19-Norcholesterol	in other cancer types (3)	no evidence	no evidence	no evidence	in other cancer types	in this cancer type and other cancer types	III-A
Cobimetinib	no evidence	no evidence	no evidence	no evidence	in this cancer type and other cancer types	in this cancer type and other cancer types	II-B

Clinical Trial Summary – *NRAS* mutation (RTK/RAS/MAPK)			
**NCT ID**	**Subject**	**phase**	**Results**
NCT04931342	BOUQUET: A Phase II, Open-Label, Multicenter, Platform Study Evaluating the Efficacy and Safety of Biomarker-Driven Therapies in Patients With Persistent or Recurrent Rare Epithelial Ovarian Tumors	II	2026
NCT02465060	MATCH: phase II trial to evaluate the proportion of patients with objective response (OR) to targeted study agent(s) in patients with advanced refractory cancers/lymphomas/multiple myeloma	II	2025
NCT02925234	DRUP: This is a prospective, non-randomized clinical trial that aims to describe the efficacy and toxicity of commercially available, **targeted anticancer drugs** prescribed for treatment of patients with **advanced cancer** with a **potentially actionable** variant as revealed by a genomic or protein expression test.	II	2027

Relevant therapy summary – *BRAF* mutation (RTK/RAS/MAPK)							
**Relevant therapy**	**OncoKB**	**FDA**	**NCCN**	**EMA**	**Clinical trials**	**Pre-clinical**	**ESCAT score**
Dabrafenib + Trametinib (V600E)	in this cancer type and other cancer types (1)	in this cancer type and other cancer types	in this cancer type and other cancer types	in other cancer types	in this cancer type and other cancer types	in this cancer type and other cancer types	II-B
Atezolizumab + Cobimetinib + Vemurafenib (V600E)	in other cancer types (1)	in other cancer types	in other cancer types	in other cancer types	in this cancer type and other cancer types	in this cancer type and other cancer types	II-B
Encorafenib + cetuximab (V600E)	in other cancer types (1)	in other cancer types	in other cancer types	in other cancer types	in other cancer types	in this cancer type and other cancer types	II-B
Vemurafenib (V600)	in other cancer types (1)	no evidence	in other cancer types	in other cancer types	in this cancer type and other cancer types	in this cancer type and other cancer types	II-B
Dabrafenib (V600)	in other cancer types (1)	no evidence	no evidence	in other cancer types	in this cancer type and other cancer types	in this cancer type and other cancer types	II-B
regorafenib	no evidence	no evidence	no evidence	no evidence	in this cancer type and other cancer types	in this cancer type and other cancer types	II-B
Encorafenib + Binimetinib (V600)	in other cancer types (1)	in other cancer types	in other cancer types	in other cancer types	in other cancer types	in this cancer type and other cancer types	II-B
Trametinib (V600E, V600K)	in other cancer types (1)	no evidence	no evidence	in other cancer types	in this cancer type and other cancer types	in this cancer type and other cancer types	II-B
Vemurafenib + Cobimetinib (V600)	in this cancer type (4)	in other cancer types	in other cancer types	in other cancer types	in this cancer type and other cancer types	in this cancer type and other cancer types	II-B
Trametinib, Cobimetinib (fusions)	in other cancer types (2)	in other cancer types	in other cancer types	no evidence	in this cancer type and other cancer types	in this cancer type and other cancer types	II-B

in this cancer type (green) | in other cancer types (orange) | in this cancer type and other cancer types (blue) | no evidence (grey)

Clinical Trial Summary – *BRAF* mutation (RTK/RAS/MAPK)

NCT ID	Subject	phase	Results
NCT04931342	A Phase II, Open-Label, Multicenter, Platform Study Evaluating the Efficacy and Safety of Biomarker-Driven Therapies in Patients With Persistent or Recurrent Rare Epithelial Ovarian Tumors	II	2026
NCT02925234	DRUP: This is a prospective, non-randomized clinical trial that aims to describe the efficacy and toxicity of commercially available, **targeted anticancer drugs** prescribed for treatment of patients with **advanced cancer** with a **potentially actionable** variant as revealed by a genomic or protein expression test.	II	2027
NCT02465060	MATCH: phase II trial to evaluate the proportion of patients with objective response (OR) to targeted study agent(s) in patients with advanced refractory cancers/lymphomas/multiple myeloma	II	2024
NCT02693535	TAPUR: **Testing** the Use of Food and Drug Administration (FDA) **Approved Drugs** That **Target** a Specific **Abnormality** in a **Tumor Gene** in People With Advanced Stage **Cancer (TAPUR)**	II	2025
NCT05768178	DETERMINE: Treatment arm 05 (vemurafenib and Cobimetinib) aims to evaluate the efficacy of vemurafenib and cobimetinib in adult patients with rare* cancers with BRAF V600 mutations or in common cancers where BRAFV600 mutations and considered to be infrequent.	II/III	2029

Relevant therapy summary – *FGFR1* mutation (RTK/RAS/MAPK)

Relevant therapy	OncoKB	FDA	NCCN	EMA	Clinical trials	Pre-clinical	ESCAT score
Pemigatinib	in other cancer types [1]	in other cancer types	in other cancer types	in other cancer types	in other cancer types	no evidence	III-A
Erdafitinib, AZD4547	in this cancer type and other cancer types	in other cancer types	in other cancer types	no evidence	in this cancer type and other cancer types	no evidence	III-A
Surufatinib (+ pamiparib)	no evidence	no evidence	no evidence	no evidence	in this cancer type	no evidence	III-A
Infigratinib (K656E)	no evidence	no evidence	no evidence	no evidence	in other cancer types	in this cancer type and other cancer types	III-A

Clinical Trial Summary – *FGFR1* mutation (RTK/RAS/MAPK)

NCT ID	Subject	phase	Results
NCT05652283	Pamiparib in Combination With **Surufatinib** in Patients With Platinum-resistant **ovarian cancer** Who Received Prior Poly (ADP-ribose) Polymerase (PARP) Inhibitors: a Multicenter, Single-arm, Phase Ib/II Trial	II	2026
NCT05019794	**Infigratinib** in Subjects With GC or GEJ With FGFR2 Amplification or Other Solid Tumors With Other FGFR Alterations (FGFR)	II	2023
NCT04096417	A Phase II, Multicenter, Single-Arm Study of **Pemigatinib** in Patients With Metastatic or Unresectable Colorectal Cancer Harboring FGFR Alterations	II	2025

Relevant therapy summary – *FGFR2* mutation (RTK/RAS/MAPK)

Relevant therapy	OncoKB	FDA	NCCN	EMA	Clinical trials	Pre-clinical	ESCAT score
sunitinib	no evidence	no evidence	no evidence	no evidence	in this cancer type and other cancer types	in other cancer types	III-A
erdafitinib	in this cancer type and other cancer types [4]	in other cancer types	in other cancer types	no evidence	in this cancer type and other cancer types	in other cancer types	III-A
Infigratinib	no evidence	in other cancer types	in other cancer types	no evidence	in other cancer types	in other cancer types	III-A
Pemigatinib	in other cancer types [1]	in other cancer types	in other cancer types	in other cancer types	in other cancer types	no evidence	III-A
AZD4547	in this cancer type and other cancer types [4]	no evidence	no evidence	no evidence	in this cancer type and other cancer types	in this cancer type and other cancer types	III-A
RLY-4008	in this cancer type and other cancer types [4]	no evidence	no evidence	no evidence	in this cancer type and other cancer types	in this cancer type and other cancer types	III-A

● in this cancer type ● in other cancer types ● in this cancer type and other cancer types ● no evidence

Clinical Trial Summary – *FGFR2* mutation (RTK/RAS/MAPK)

NCT ID	Subject	phase	Results
NCT02693535	**TAPUR: Testing** the Use of Food and Drug Administration (FDA) **Approved Drugs** That **Target** a Specific **Abnormality** in a **Tumor Gene** in People With Advanced Stage **Cancer (TAPUR)**	II	2025
NCT02925234	DRUP: This is a prospective, non-randomized clinical trial that aims to describe the efficacy and toxicity of commercially available, **targeted anticancer drugs** prescribed for treatment of patients with **advanced cancer** with a **potentially actionable** variant as revealed by a genomic or protein expression test.	II	2027
NCT02465060	MATCH: phase II trial to evaluate the proportion of patients with objective response (OR) to targeted study agent(s) in patients with advanced refractory cancers/lymphomas/multiple myeloma	II	2024
NCT05019794	A Phase IIa of **Infigratinib** in Subjects With Locally Advanced or Metastatic Gastric Cancer or Gastroesophageal Junction Adenocarcinoma With **FGFR2** Amplification or Other Advanced Solid Tumors With Other FGFR Alterations	IIa	2023
NCT02965378	**Lung-MAP**: **AZD4547** as Second-Line Therapy in Treating **FGFR** Positive Patients With Recurrent Stage IV Squamous Cell Lung Cancer	II/III	2019
NCT05086666	A Phase 1b/2 Clinical Study to Evaluate the Safety and Tolerability of **AZD4547** in Patients With Solid Tumors and Its Efficacy in Patients With Locally Advanced or Metastatic Urothelial Carcinoma With **FGFR2/3** Gene Alterations	Ib/II	2024
NCT05565794	A Phase II Study of **Pemigatinib** After Curative Local Therapy in Locally Advanced Intrahepatic Cholangiocarcinoma (iCCA) Harboring **FGFR2** Fusions/Rearrangements	II	2026
**NCT04526106**	REFOCUS: A First-in-Human Study of Highly Selective **FGFR2** Inhibitor, **RLY-4008**, in Patients With ICC and Other Advanced Solid Tumors	I/II	2024
NCT04096417	A Phase II, Multicenter, Single-Arm Study of **Pemigatinib** in Patients With Metastatic or Unresectable Colorectal Cancer Harboring FGFR Alterations	II	2025

Relevant therapy summary – *FGFR2* amplification (RTK/RAS/MAPK)

Relevant therapy	OncoKB	FDA	NCCN	EMA	Clinical trials	Pre-clinical	ESCAT score
sunitinib	no evidence	no evidence	no evidence	no evidence	in this cancer type and other cancer types	in other cancer types	III-A
RLY-4008	in this cancer type and other cancer types [4]	no evidence	no evidence	no evidence	in this cancer type and other cancer types	in this cancer type and other cancer types	III-A
Pemigatinib	in other cancer types [1]	in other cancer types	in other cancer types	in other cancer types	in other cancer types	no evidence	III-A

Clinical Trial Summary – *FGFR2* amplification (RTK/RAS/MAPK)

NCT ID	Subject	phase	Results
NCT02925234	DRUP: This is a prospective, non-randomized clinical trial that aims to describe the efficacy and toxicity of commercially available, **targeted anticancer drugs** prescribed for treatment of patients with **advanced cancer** with a **potentially actionable** variant as revealed by a genomic or protein expression test.	II	2027
**NCT04526106**	REFOCUS: A First-in-Human Study of Highly Selective **FGFR2** Inhibitor, **RLY-4008**, in Patients With ICC and Other Advanced Solid Tumors	I/II	2024
NCT02693535	**TAPUR: Testing** the Use of Food and Drug Administration (FDA) **Approved Drugs** That **Target** a Specific **Abnormality** in a **Tumor Gene** in People With Advanced Stage **Cancer (TAPUR)**	II	2025
NCT05565794	A Phase II Study of **Pemigatinib** After Curative Local Therapy in Locally Advanced Intrahepatic Cholangiocarcinoma (iCCA) Harboring **FGFR2** Fusions/Rearrangements	II	2026
NCT04096417	A Phase II, Multicenter, Single-Arm Study of **Pemigatinib** in Patients With Metastatic or Unresectable Colorectal Cancer Harboring FGFR Alterations	II	2025

green: in this cancer type | orange: in other cancer types | blue: in this cancer type and other cancer types | grey: no evidence

Relevant therapy summary – *FGFR3* mutation (RTK/RAS/MAPK)

Relevant therapy	OncoKB	FDA	NCCN	EMA	Clinical trials	Pre-clinical	ESCAT score
Erdafitinib (G370C, R248C, S249C, Y373C)	orange [1]	orange	orange	orange	orange	blue	III-A
Erdafitinib (G380R, K650, S371C)	orange [3]	grey	grey	orange	orange	blue	III-A
Erdafitinib, AZD4547	orange [4]	grey	grey	orange	orange	blue	III-A
sunitinib	grey	grey	grey	grey	blue	orange	III-A
vofatamab	grey	grey	grey	grey	blue	orange	III-A
TYRA-300	grey	grey	grey	grey	blue	orange	III-A
Lenvatinib	grey	grey	grey	orange	orange	orange	III-A
Pemigatinib	orange [1]	orange	orange	orange	orange	blue	III-A
ponatinib	grey	grey	grey	grey	blue	orange	III-A

Clinical Trial Summary – *FGFR3* mutation (RTK/RAS/MAPK)

NCT ID	Subject	phase	Results
NCT05565794	A Phase II Study of **Pemigatinib** After Curative Local Therapy in Locally Advanced Intrahepatic Cholangiocarcinoma (iCCA) Harboring **FGFR2** Fusions/Rearrangements	II	2026
NCT02925234	DRUP: This is a prospective, non-randomized clinical trial that aims to describe the efficacy and toxicity of commercially available, **targeted anticancer drugs** prescribed for treatment of patients with **advanced cancer** with a **potentially actionable** variant as revealed by a genomic or protein expression test.	II	2027
NCT02693535	**TAPUR: Testing** the Use of Food and Drug Administration (FDA) **Approved Drugs** That **Target** a Specific **Abnormality** in a **Tumor Gene** in People With Advanced Stage **Cancer (TAPUR)**	II	2025
NCT05544552	A Multicenter, Open-label Phase 1/2 Study of TYRA300 in Advanced Urothelial Carcinoma and Other Solid Tumors With Activating FGFR3 Gene Alterations (SURF301)	I/II	2027
NCT05363605	A Phase 1/2 Study of [225Ac]-FPI-1966, [111In]-FPI-1967, and Vofatamab in Participants With **FGFR3**-expressing Advanced, Inoperable, Metastatic and/or Recurrent Solid Tumours	I/II	2026
NCT04917809	A Phase 2 "Window of Opportunity" Trial of Targeted Therapy With **Erdafitinib** in Patients With Recurrent **FGFR3**-Altered Non-Muscle Invasive Bladder Cancer	II	2025
NCT02272998	Phase II Study of **Ponatinib** for Advanced Cancers With Genomic Alterations in Fibroblastic Growth Factor Receptor (**FGFR**) and Other Genomic Targets (KIT, PDGF, RET FLT3, ABL1)	II	2023

Relevant therapy summary – *FGFR3* amplification (RTK/RAS/MAPK)

Relevant therapy	OncoKB	FDA	NCCN	EMA	Clinical trials	Pre-clinical	ESCAT score
ponatinib	grey	grey	grey	grey	blue	orange	III-A
Pemigatinib	orange [1]	orange	orange	orange	orange	blue	III-A
Erdafitinib	grey	orange	orange	orange	orange	blue	III-A
sunitinib	grey	grey	grey	grey	blue	orange	III-A
Lenvatinib	grey	grey	grey	orange	orange	orange	III-A

🟢 in this cancer type 🟠 in other cancer types 🔵 in this cancer type and other cancer types ⊘ no evidence

Clinical Trial Summary – *FGFR3* amplification (RTK/RAS/MAPK)

NCT ID	Subject	phase	Results
NCT02925234	DRUP: This is a prospective, non-randomized clinical trial that aims to describe the efficacy and toxicity of commercially available, **targeted anticancer drugs** prescribed for treatment of patients with **advanced cancer** with a **potentially actionable** variant as revealed by a genomic or protein expression test.	II	2027
NCT04917809	A Phase 2 "Window of Opportunity" Trial of Targeted Therapy With **Erdafitinib** in Patients With Recurrent **FGFR3**-Altered Non-Muscle Invasive Bladder Cancer	II	2025
NCT02272998	Phase II Study of **Ponatinib** for Advanced Cancers With Genomic Alterations in Fibroblastic Growth Factor Receptor (**FGFR**) and Other Genomic Targets (KIT, PDGF, RET FLT3, ABL1)	II	2023
NCT05544552	A Multicenter, Open-label Phase 1/2 Study of TYRA300 in Advanced Urothelial Carcinoma and Other Solid Tumors With Activating FGFR3 Gene Alterations (SURF301)	I/II	2027
NCT05363605	A Phase 1/2 Study of [225Ac]-FPI-1966, [111In]-FPI-1967, and Vofatamab in Participants With **FGFR3**-expressing Advanced, Inoperable, Metastatic and/or Recurrent Solid Tumours	I/II	2026
NCT02693535	**TAPUR: Testing** the Use of Food and Drug Administration (FDA) **Approved Drugs** That **Target** a Specific **Abnormality** in a **Tumor Gene** in People With Advanced Stage **Cancer** (TAPUR)	II	2025

Relevant therapy summary – *MET* mutation (RTK/RAS/MAPK)

Relevant therapy	OncoKB	FDA	NCCN	EMA	Clinical trials	Pre-clinical	ESCAT score
Capmatinib (D1010, Ex 14 Del, Ex 14 in-frame del, Ex 14 splice mut)	🟠 1	🟠	🟠	🟠	🟠	🔵	III-A
Tepotinib (D1010, Ex 14 Del, Ex 14 in-frame del, Ex 14 splice mut)	🟠 1	🟠	🟠	🟠	🟠	🔵	III-A
Crizotinib	🟠 2	⊘	🟠	⊘	🔵	🔵	III-A

Clinical Trial Summary – *MET* mutation (RTK/RAS/MAPK)

NCT ID	Subject	phase	Results
NCT02925234	DRUP: This is a prospective, non-randomized clinical trial that aims to describe the efficacy and toxicity of commercially available, **targeted anticancer drugs** prescribed for treatment of patients with **advanced cancer** with a **potentially actionable** variant as revealed by a genomic or protein expression test.	II	2027
NCT02693535	**TAPUR: Testing** the Use of Food and Drug Administration (FDA) **Approved Drugs** That **Target** a Specific **Abnormality** in a **Tumor Gene** in People With Advanced Stage **Cancer** (TAPUR)	II	2025
NCT02465060	**MATCH:** phase II trial to evaluate the proportion of patients with objective response (OR) to targeted study agent(s) in patients with advanced refractory cancers/lymphomas/multiple myeloma	II	2024

Relevant therapy summary – *MET* amplification (RTK/RAS/MAPK)

Relevant therapy	OncoKB	FDA	NCCN	EMA	Clinical trials	Pre-clinical	ESCAT score
Capmatinib	🟠 2	🟠	🟠	🟠	🟠	⊘	III-A
Crizotinib	🟠 2	🟠	🟠	⊘	🟠	⊘	III-A
Tepotinib	🟠 2	🟠	🟠	🟠	🟠	⊘	III-A
Telisotuzumab vedotin	🟠 3	⊘	🟠	⊘	🟠	⊘	III-A

in this cancer type | in other cancer types | in this cancer type and other cancer types | no evidence

Clinical Trial Summary – *MET* amplification (RTK/RAS/MAPK)			
**NCT ID**	**Subject**	**phase**	**Results**
NCT03539536	Phase 2, Open-Label Safety and Efficacy Study of **Telisotuzumab Vedotin** (ABBV-399) in Subjects With Previously Treated c-**Met+ Non-Small Cell Lung Cancer**	II	2025
NCT02925234	DRUP: This is a prospective, non-randomized clinical trial that aims to describe the efficacy and toxicity of commercially available, **targeted anticancer drugs** prescribed for treatment of patients with **advanced cancer** with a **potentially actionable** variant as revealed by a genomic or protein expression test.	II	2027
NCT02693535	**TAPUR: Testing** the Use of Food and Drug Administration (FDA) **Approved Drugs** That **Target** a Specific **Abnormality** in a **Tumor Gene** in People With Advanced Stage **Cancer** (TAPUR)	II	2025
NCT02465060	**MATCH:** phase II trial to evaluate the proportion of patients with objective response (OR) to targeted study agent(s) in patients with advanced refractory cancers/lymphomas/multiple myeloma	II	2024

Relevant therapy summary – *CCND1* mutation (cell cycle)							
**Relevant therapy**	**OncoKB**	**FDA**	**NCCN**	**EMA**	**Clinical trials**	**Pre-clinical**	**ESCAT score**
Palbociclib (RB+ on IHC)	no evidence	no evidence	no evidence	no evidence	in this cancer type and other cancer types	no evidence	IV-B
Abemaciclib	no evidence	no evidence	no evidence	no evidence	in this cancer type and other cancer types	no evidence	IV-B

Clinical Trial Summary – CCND1 mutation (cell cycle)			
**NCT ID**	**Subject**	**phase**	**Results**
NCT02925234	DRUP: This is a prospective, non-randomized clinical trial that aims to describe the efficacy and toxicity of commercially available, targeted anticancer drugs prescribed for treatment of patients with advanced cancer with a potentially actionable variant as revealed by a genomic or protein expression test.	II	2027
NCT02465060	**MATCH:** phase II trial to evaluate the proportion of patients with objective response (OR) to targeted study agent(s) in patients with advanced refractory cancers/lymphomas/multiple myeloma	II	2024

Relevant therapy summary – *CCND1* amplification (cell cycle)							
**Relevant therapy**	**OncoKB**	**FDA**	**NCCN**	**EMA**	**Clinical trials**	**Pre-clinical**	**ESCAT score**
Palbociclib (RB+ on IHC)	no evidence	no evidence	no evidence	no evidence	in this cancer type and other cancer types	no evidence	IV-B
Abemaciclib	no evidence	no evidence	no evidence	no evidence	in this cancer type and other cancer types	no evidence	IV-B

Clinical Trial Summary – *CCND1* amplification (cell cycle)			
**NCT ID**	**Subject**	**phase**	**Results**
NCT02465060	**MATCH:** phpase II trial to evaluate the proportion of patients with objective response (OR) to targeted study agent(s) in patients with advanced refractory cancers/lymphomas/multiple myeloma	II	2025
NCT02925234	DRUP: This is a prospective, non-randomized clinical trial that aims to describe the efficacy and toxicity of commercially available, **targeted anticancer drugs** prescribed for treatment of patients with **advanced cancer** with a **potentially actionable** variant as revealed by a genomic or protein expression test.	II	2027
NCT02693535	**TAPUR: Testing** the Use of Food and Drug Administration (FDA) **Approved Drugs** That **Target** a Specific **Abnormality** in a **Tumor Gene** in People With Advanced Stage **Cancer** (TAPUR)	II	2025

Pre - clinical Research Summary – *CCND1* amplification (cell cycle)		
**Title**	**model**	**journal**
MicroRNA-195 suppresses cell proliferation, migration and invasion in epithelial ovarian carcinoma via inhibition of the CDC42/CCND1 pathway[44]	*In vitro*	Int. J. of Molecular Medicine
Cyclin D1 silencing impairs DNA double strand break repair, sensitizes BRCA1 wildtype ovarian cancer cells to olaparib	*In vitro*	Gynecologic Oncology

🟢 in this cancer type 🟠 in other cancer types 🔵 in this cancer type and other cancer types ⚪ no evidence

Relevant therapy summary – *CDKN2A* mutation (cell cycle)							
**Relevant therapy**	**OncoKB**	**FDA**	**NCCN**	**EMA**	**Clinical trials**	**Pre-clinical**	**ESCAT score**
Palbociclib	🔵 [4]	⚪	⚪	⚪	🔵	🔵	II-B
Abemaciclib	🔵 [4]	⚪	⚪	⚪	🔵	🔵	II-B
Ribociclib	🔵 [4]	⚪	⚪	⚪	🔵	🔵	II-B
Ilorasertib	⚪	⚪	⚪	⚪	🔵	🔵	II-B

Clinical Trial Summary – *CDKN2A* mutation (cell cycle)			
**NCT ID**	**Subject**	**phase**	**Results**
NCT02925234	DRUP: This is a prospective, non-randomized clinical trial that aims to describe the efficacy and toxicity of commercially available, **targeted** anticancer drugs prescribed for treatment of patients with advanced cancer with a potentially **actionable variant** as revealed by a genomic or protein expression test.	II	2027
NCT02693535	**TAPUR: Testing** the Use of Food and Drug Administration (FDA) **Approved Drugs** That **Target** a Specific **Abnormality** in a **Tumor Gene** in People With Advanced Stage **Cancer** (TAPUR)	II	2025
NCT02478320	A Proof-of-Concept Study for **Ilorasertib** (ABT-348) Activity in Patients With CDKN2A-Deficient Advanced Solid Cancers: a Phase II Basket Trial	II	2023
NCT03297606	CAPTUR: Canadian Profiling and Targeted Agent Utilization Trial: A Phase II Basket Trial	II	2027

Pre - clinical Research Summary – CDKN2A mutation (cell cycle)		
**Title**	**model**	**journal**
Durable response to palbociclib and letrozole in ovarian cancer with CDKN2A loss [45]	*Case study*	Cancer biology and Therapy

Relevant therapy summary – *SMARCA4* mutation							
**Relevant therapy**	**OncoKB**	**FDA**	**NCCN**	**EMA**	**Clinical trials**	**Pre-clinical**	**ESCAT score**
PRT3789	⚪	⚪	⚪	⚪	🔵	🔵	II-B
palbociclib	⚪	⚪	⚪	⚪	🔵	🔵	II-B
atezolizumab	⚪	⚪	⚪	⚪	🔵	🔵	II-B

Clinical Trial Summary – *SMARCA4* mutation			
**NCT ID**	**Subject**	**phase**	**Results**
NCT05639751	A Phase 1 Open-Label, Multi-Center, Safety and Efficacy Study of PRT3789 in Participants With Select Advanced or Metastatic Solid Tumors With a SMARCA4 Mutation	I	2026
NCT02925234	DRUP: This is a prospective, non-randomized clinical trial that aims to describe the efficacy and toxicity of commercially available, **targeted anticancer drugs** prescribed for treatment of patients with **advanced cancer** with a **potentially actionable** variant as revealed by a genomic or protein expression test.	II	2027
NCT05286801	A Phase 1/2 Study of Tiragolumab (NSC# 827799) and Atezolizumab (NSC# 783608) in Patients With Relapsed or Refractory SMARCB1 or SMARCA4 Deficient Tumors	I/II	2025

Relevant therapy summary – *SMARCB1* mutation							
**Relevant therapy**	**OncoKB**	**FDA**	**NCCN**	**EMA**	**Clinical trials**	**Pre-clinical**	**ESCAT score**
Tazemetostat (deletion)	🟠 [1]	🟠	⚪	⚪	🟠	🔵	II-B
atezolizumab	⚪	⚪	⚪	⚪	🔵	🔵	II-B

in this cancer type | in other cancer types | in this cancer type and other cancer types | no evidence

Clinical Trial Summary – *SMARCB1* mutation			
**NCT ID**	**Subject**	**phase**	**Results**
NCT05286801	A Phase 1/2 Study of Tiragolumab (NSC# 827799) and Atezolizumab (NSC# 783608) in Patients With Relapsed or Refractory SMARCB1 or SMARCA4 Deficient Tumors	I/II	2025

Relevant therapy summary – *GNAS* mutation							
**Relevant therapy**	**OncoKB**	**FDA**	**NCCN**	**EMA**	**Clinical trials**	**Pre-clinical**	**ESCAT score**
trametinib	no evidence	no evidence	no evidence	no evidence	in this cancer type and other cancer types	no evidence	X

Clinical Trial Summary – *GNAS* mutation			
**NCT ID**	**Subject**	**phase**	**Results**
NCT02925234	DRUP: This is a prospective, non-randomized clinical trial that aims to describe the efficacy and toxicity of commercially available, **targeted anticancer drugs** prescribed for treatment of patients with **advanced cancer** with a **potentially actionable** variant as revealed by a genomic or protein expression test.	II	2027

Relevant therapy summary – *POLE* mutation							
**Relevant therapy**	**OncoKB**	**FDA**	**NCCN**	**EMA**	**Clinical trials**	**Pre-clinical**	**ESCAT score**
pembroluzimab	no evidence	no evidence	no evidence	no evidence	in this cancer type and other cancer types	no evidence	X
toripalimab	no evidence	no evidence	no evidence	no evidence	in this cancer type and other cancer types	no evidence	X

Clinical Trial Summary – *POLE* mutation			
**NCT ID**	**Subject**	**phase**	**Results**
NCT03428802	A Basket Trial of Pembrolizumab in Patients With Advanced Solid Tumors and Genomic Instability	II	2023
NCT03810339	A Phase II Open Label Study of Toripalimab, a PD-1 Antibody, in Participants With POLE or POLD-1 Mutated and Non-MSI-H Advanced Solid Tumors	II	2024

References

1. Leijen, S. *et al.* Phase II Study of WEE1 Inhibitor AZD1775 Plus Carboplatin in Patients With TP53-Mutated Ovarian Cancer Refractory or Resistant to First-Line Therapy Within 3 Months. *J Clin Oncol* **34**, 4354–4361 (2016).
2. Westin, S. N. *et al.* EFFORT: EFFicacy Of adavosertib in parp ResisTance: A randomized two-arm non-comparative phase II study of adavosertib with or without olaparib in women with PARP-resistant ovarian cancer. *https://doi.org/10.1200/JCO.2021.39.15_suppl.5505* **19**, 14–15 (2021).
3. Fransson, Å. *et al.* Strong synergy with APR-246 and DNA-damaging drugs in primary cancer cells from patients with TP53 mutant High-Grade Serous ovarian cancer. *J Ovarian Res* **9**, 1–10 (2016).
4. Gonzalez Martin, A. *et al.* A phase III, randomized, double blinded trial of platinum based chemotherapy with or without atezolizumab followed by niraparib maintenance with or without atezolizumab in patients with recurrent ovarian, tubal, or peritoneal cancer and platinum treatment free interval of more than 6 months: ENGOT-Ov41/GEICO 69-O/ANITA Trial. *Int J Gynecol Cancer* **31**, 617–622 (2021).
5. Elyashiv, O. *et al.* ICON 9-an international phase III randomized study to evaluate the efficacy of maintenance therapy with olaparib and cediranib or olaparib alone in patients with relapsed platinum-sensitive ovarian cancer following a response to platinum-based chemotherapy. *Int J Gynecol Cancer* **31**, 134–138 (2021).
6. Ray-Coquard, I. *et al.* Olaparib plus Bevacizumab as First-Line Maintenance in Ovarian Cancer. *N Engl J Med* **381**, 2416–2428 (2019).
7. Fujiwara, K. *et al.* Olaparib plus bevacizumab as maintenance therapy in patients with newly diagnosed, advanced ovarian cancer: Japan subset from the PAOLA-1/ENGOT-ov25 trial. *J Gynecol Oncol* **32**, (2021).
8. González-Martín, A. *et al.* Niraparib in Patients with Newly Diagnosed Advanced Ovarian Cancer. *N Engl J Med* **381**, 2391–2402 (2019).
9. Lorusso, D. *et al.* Feasibility Study of a Network Meta-Analysis and Unanchored Population-Adjusted Indirect Treatment Comparison of Niraparib, Olaparib, and Bevacizumab as Maintenance Therapies in Patients with Newly Diagnosed Advanced Ovarian Cancer. *Cancers (Basel)* **14**, (2022).
10. Barretina-Ginesta, M.-P. *et al.* Quality-adjusted time without symptoms of disease or toxicity and quality-adjusted progression-free survival with niraparib maintenance in first-line ovarian cancer in the PRIMA trial. *Ther Adv Med Oncol* **14**, 175883592211261 (2022).
11. O'Cearbhaill, R. E. *et al.* Efficacy of niraparib by time of surgery and postoperative residual disease status: A post hoc analysis of patients in the PRIMA/ENGOT-OV26/GOG-3012 study. *Gynecol Oncol* **166**, 36–43 (2022).
12. Moore, K. *et al.* Maintenance Olaparib in Patients with Newly Diagnosed Advanced Ovarian Cancer. *N Engl J Med* **379**, 2495–2505 (2018).
13. Tattersall, A., Ryan, N., Wiggans, A. J., Rogozińska, E. & Morrison, J. Poly(ADP-ribose) polymerase (PARP) inhibitors for the treatment of ovarian cancer. *Cochrane Database Syst Rev* **2**, (2022).
14. Banerjee, S. *et al.* Maintenance olaparib for patients with newly diagnosed advanced ovarian cancer and a BRCA mutation (SOLO1/GOG 3004): 5-year follow-up of a randomised, double-blind, placebo-controlled, phase 3 trial. *Lancet Oncol* **22**, 1721–1731 (2021).
15. Friedlander, M. *et al.* Patient-centred outcomes and effect of disease progression on health status in patients with newly diagnosed advanced ovarian cancer and a BRCA mutation receiving maintenance olaparib or placebo (SOLO1): a randomised, phase 3 trial. *Lancet Oncol* **22**, 632–642 (2021).
16. Mahdi, H. *et al.* Ceralasertib-Mediated ATR Inhibition Combined With Olaparib in Advanced Cancers Harboring DNA Damage Response and Repair Alterations (Olaparib Combinations). *https://doi.org/10.1200/PO.20.00439* 1432–1442 (2021) doi:10.1200/PO.20.00439.
17. Wilson, Z. *et al.* ATR Inhibitor AZD6738 (Ceralasertib) Exerts Antitumor Activity as a Monotherapy and in Combination with Chemotherapy and the PARP Inhibitor Olaparib. *Cancer Res* **82**, 1140–1152 (2022).
18. Smith, G., Alholm, Z., Coleman, R. L. & Monk, B. J. DNA Damage Repair Inhibitors-Combination Therapies. *Cancer J* **27**, 501–505 (2021).
19. ali, azka *et al.* Phase II trial of the PARP inhibitor, niraparib, in BAP1 and other DNA damage response (DDR) pathway deficient neoplasms (NCT03207347). *https://doi.org/10.1200/JCO.2020.38.15_suppl.e22061* **38**, e22061–e22061 (2020).
20. Keane, F., Park, W. & O'Reilly, E. M. Homologous Recombination Deficiency in Pancreatic Cancer: Poly (ADP-ribose) Polymerase Inhibition, Checkpoint Inhibition, or a Combination of Both? *https://doi.org/10.1200/PO.22.00141* (2022) doi:10.1200/PO.22.00141.
21. Lücking, U. *et al.* Damage Incorporated: Discovery of the Potent, Highly Selective, Orally Available ATR Inhibitor BAY 1895344 with Favorable Pharmacokinetic Properties and Promising Efficacy in Monotherapy and in Combination Treatments in Preclinical Tumor Models. *J Med Chem* **63**, 7293–7325 (2020).
22. Yap, T. A. *et al.* First-in-human trial of the oral ataxia telangiectasia and rad3-related (Atr) inhibitor bay 1895344 in patients with advanced solid tumors. *Cancer Discov* **11**, 80–91 (2021).
23. Fennell, D. A. *et al.* Rucaparib in patients with BAP1-deficient or BRCA1-deficient mesothelioma (MiST1): an open-label, single-arm, phase 2a clinical trial. *Lancet Respir Med* **9**, 593–600 (2021).
24. Han, A. *et al.* Pyruvate dehydrogenase inactivation causes glycolytic phenotype in BAP1 mutant uveal melanoma. *Oncogene* **41**, 1129 (2022).
25. Dudnik, E. *et al.* BAP1-Altered Malignant Pleural Mesothelioma: Outcomes With Chemotherapy, Immune Check-Point Inhibitors and Poly(ADP-Ribose) Polymerase Inhibitors. *Front Oncol* **11**, 29 (2021).
26. Mandal, J., Mandal, P., Wang, T. L. & Shih, I. M. Treating ARID1A mutated cancers by harnessing synthetic lethality and DNA damage response. *J Biomed Sci* **29**, 71 (2022).
27. Takahashi, K., Takenaka, M., Okamoto, A., Bowtell, D. D. L. & Kohno, T. Treatment Strategies for ARID1A-Deficient Ovarian Clear Cell Carcinoma. *Cancers (Basel)* **13**, (2021).
28. Campo, E. *et al.* The 2008 WHO classification of lymphoid neoplasms and beyond: evolving concepts and practical applications. *Blood* **117**, 5019–5032 (2011).

29. Sen, M. *et al.* ARID1A facilitates KRAS signaling-regulated enhancer activity in an AP1-dependent manner in colorectal cancer cells. *Clin Epigenetics* **11**, (2019).
30. Yamada, L. *et al.* Selective sensitivity of EZH2 inhibitors based on synthetic lethality in ARID1A-deficient gastric cancer. *Gastric Cancer* **24**, 60–71 (2021).
31. Bitler, B. G. *et al.* Synthetic lethality by targeting EZH2 methyltransferase activity in ARID1A-mutated cancers. *Nat Med* **21**, 231–238 (2015).
32. Xu, G. *et al.* ARID1A determines luminal identity and therapeutic response in estrogen-receptor-positive breast cancer. *Nat Genet* **52**, 198–207 (2020).
33. Cheng, X., Zhao, J. X., Dong, F. & Cao, X. C. ARID1A Mutation in Metastatic Breast Cancer: A Potential Therapeutic Target. *Front Oncol* **11**, (2021).
34. Banerjee, S. *et al.* ATARI trial: ATR inhibitor in combination with olaparib in gynecological cancers with ARID1A loss or no loss (ENGOT/GYN1/NCRI). *International Journal of Gynecologic Cancer* **31**, 1471–1475 (2021).
35. Lakhani, N. J. *et al.* Phase 1/2 first-in-human (FIH) study of CPI-0209, a novel small molecule inhibitor of enhancer of zeste homolog 2 (EZH2) in patients with advanced tumors. *https://doi.org/10.1200/JCO.2021.39.15_suppl.3104* **39**, 3104–3104 (2021).
36. Park, Y. *et al.* Loss of ARID1A in tumor cells renders selective vulnerability to combined ionizing radiation and PARP inhibitor therapy. *Clinical Cancer Research* **25**, 5584–5593 (2019).
37. Banerji, U. *et al.* A Phase I Open-Label Study to Identify a Dosing Regimen of the Pan-AKT Inhibitor AZD5363 for Evaluation in Solid Tumors and in PIK3CA-Mutated Breast and Gynecologic Cancers. *Clin Cancer Res* **24**, 2050–2059 (2018).
38. Smyth, L. M. *et al.* Capivasertib, an AKT Kinase Inhibitor, as Monotherapy or in Combination with Fulvestrant in Patients with AKT1 E17K-Mutant, ER-Positive Metastatic Breast Cancer. *Clin Cancer Res* **26**, 3947–3957 (2020).
39. Wu, Y. H., Huang, Y. F., Chen, C. C., Huang, C. Y. & Chou, C. Y. Comparing PI3K/Akt Inhibitors Used in Ovarian Cancer Treatment. *Front Pharmacol* **11**, (2020).
40. Konstantinopoulos, P. A. *et al.* EPIK-O/ENGOT-OV61: Alpelisib plus olaparib vs cytotoxic chemotherapy in high-grade serous ovarian cancer (phase III study). *https://doi.org/10.2217/fon-2022-0666* (2022) doi:10.2217/FON-2022-0666.
41. Meng, D. *et al.* Development of PI3K inhibitors: Advances in clinical trials and new strategies (Review). *Pharmacol Res* **173**, 105900 (2021).
42. Mensah, F. A., Blaize, J. P. & Bryan, L. J. Spotlight on copanlisib and its potential in the treatment of relapsed/refractory follicular lymphoma: evidence to date. *Onco Targets Ther* **11**, 4817–4827 (2018).
43. Turner, N. *et al.* Ipatasertib plus paclitaxel for PIK3CA/AKT1/PTEN-altered hormone receptor-positive HER2-negative advanced breast cancer: primary results from cohort B of the IPATunity130 randomized phase 3 trial. *Breast Cancer Res Treat* **191**, 565 (2022).
44. Hao, X. *et al.* MicroRNA-195 suppresses cell proliferation, migration and invasion in epithelial ovarian carcinoma via inhibition of the CDC42/CCND1 pathway. *Int J Mol Med* **46**, 1862–1872 (2020).
45. Frisone, D. *et al.* Durable response to palbociclib and letrozole in ovarian cancer with CDKN2A loss. *https://doi.org/10.1080/15384047.2019.1685291* **21**, 197–202 (2019).

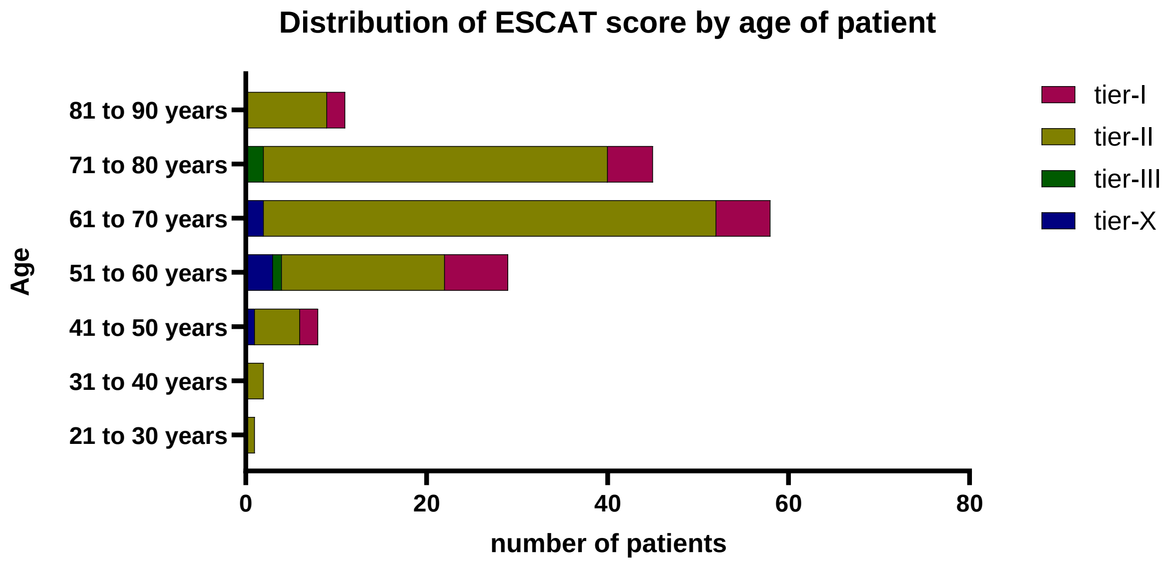

Supplementary Figure 1 – ESCAT scoring distribution by age

Supplementary Methods 1 – Detailed description of methods applied for molecular profiling

DNA extraction from FFPE biopsy material was performed using the QIAamp DNA FFPE Tissue Kit (QIAgen). For DNA extraction from peripheral blood lymphocytes we used ReliaPrep™ Large Volume HT gDNA Isolation System (Promega) before December, 2018 or Magcore® Genomic DNA Large Volume Whole Blood Kit (Code 104) (RBCBioscience) from January, 2019 on. The amplicon library was sequenced on the Miseq instrument (Illumina). Target enrichment was performed by KAPA HyperCap protocol using KAPA HyperExplore Probes (Roche), followed by sequencing on NovaSeq6000/NextSeq2000/Miseq (Illumina). NGS data were processed using an in-house bcbio pipeline including data mapping to the reference genome by BWA, variant calling using VarDict, and variant annotation in Ensembl Variant Effect Predictor and dbNSFP/dbscSNV databases. Final quality control was performed by FastQC, samtools and bcftools. Coverage was analyzed using mosdepth (minimum 300x for FFPE samples and minimum 30x for blood). In FFPE gene amplifications/deletions were based on coverage analysis. At the germline level exon spanning deletions/duplications were investigated by MLPA (P002, P045) or digital MLPA (D001), according to manufacturer's instructions (MRC Holland).

Supplementary Table 4 – Solid cancer panel version 1 with 69 genes applied to 129 patients

AKT1	*ALK*	*APC*	*AR*	*BAP1*	*BRAF*	*BRCA1*	*BRCA2*	*CCND1*
CDK4	*CDK6*	*CDKN2A*	*CDKN2B*	*CTNNB1*	*DDR2*	*DICER1*	*DPYD*	*EGFR*
ERBB2	*ERBB3*	*ERBB4*	*ESR1*	*FBXW7*	*FGFR1*	*FGFR2*	*FGFR3*	*FOXL2*
FRK	*GATA3*	*GNA11*	*GNAQ*	*GNAS*	*H3F3A*	*H3F3B*	*HIST1B3*	*HIST1H3C*
HNF1A	*HRAS*	*IDH1*	*IDH2*	*IL6ST*	*JAK1*	*JAK2*	*KIT*	*KRAS*
MAP2K1	*MET*	*NRAS*	*NTRK1*	*NTRK3*	*PDGFRA*	*PIK3CA*	*PIK3R1*	*POLE*
PTEN	*RB1*	*RET*	*RNF43*	*ROS1*	*SMAD4*	*SMARCA4*	*SMARCB1*	*SMO*
SPOP	*STAT3*	*STK11*	*TERT*	*TP53*	*VHL*			

Supplementary Table 5 – Solid cancer panel version 2 with 73 genes applied to 39 patients

AKT1	*ALK*	*APC*	*AR*	*ARID1A*	*ATM*	*BAP1*	*BRAF*	*BRCA1*
BRCA2	*CCND1*	*CDK12*	*CDK4*	*CDK6*	*CDKN2A*	*CDKN2B*	*CTNNB1*	*DICER1*
DPYD	*EGFR*	*ERBB2*	*ERBB3*	*ESR1*	*FBXW7*	*FGFR1*	*FGFR2*	*FGFR3*
FGFR4	*FOXL2*	*FRK*	*GATA3*	*GNA11*	*GNAQ*	*GNAS*	*H3F3A*	*H3F3B*
HIST1B3	*HIST1H3C*	*HNF1A*	*HRAS*	*IDH1*	*IDH2*	*IL6ST*	*JAK1*	*JAK2*
KEAP1	*KIT*	*KRAS*	*MAP2K1*	*MET*	*MYOD1*	*NRAS*	*NTRK1*	*NTRK2*
NTRK3	*PDGFRA*	*PDGFRB*	*PIK3CA*	*PIK3R1*	*POLE*	*PTEN*	*RB1*	*RET*
RNF43	*ROS1*	*SMAD4*	*SMO*	*SPOP*	*STAT3*	*STK11*	*TERT*	*TP53*
VHL								

Supplementary Table 6 – Germline HBOC cancer panel 3 with 12 genes applied to 101 patients

ATM	*BRCA1*	*BRCA2*	*BRIP1*	*CHEK2*	*MLH1*	*MSH2*	*MSH6*	*PALB2*
RAD51C	*RAD51D*	*TP53*						
