# Supplementary material for: Identification of potentially actionable genetic variants in epithelial ovarian cancer: a retrospective cohort study
Source: NPJ Precis Oncol. 2024 Mar 22;8:71. doi: 10.1038/s41698-024-00565-2 (PMC10959961; doi:10.1038/s41698-024-00565-2)
Supplement: Supplementary file 2 — REPORTING SUMMARY [file 41698_2024_565_MOESM2_ESM.pdf]

nature portfolio

Corresponding author(s): Kathleen Claes

Last updated by author(s): Feb 29, 2024

# Reporting Summary

Nature Portfolio wishes to improve the reproducibility of the work that we publish. This form provides structure for consistency and transparency in reporting. For further information on Nature Portfolio policies, see our Editorial Policies and the Editorial Policy Checklist.

## Statistics

For all statistical analyses, confirm that the following items are present in the figure legend, table legend, main text, or Methods section.

| n/a | Confirmed | |
|---|---|---|
| ☐ | ☒ | The exact sample size (*n*) for each experimental group/condition, given as a discrete number and unit of measurement |
| ☒ | ☐ | A statement on whether measurements were taken from distinct samples or whether the same sample was measured repeatedly |
| ☒ | ☐ | The statistical test(s) used AND whether they are one- or two-sided<br>*Only common tests should be described solely by name; describe more complex techniques in the Methods section.* |
| ☒ | ☐ | A description of all covariates tested |
| ☒ | ☐ | A description of any assumptions or corrections, such as tests of normality and adjustment for multiple comparisons |
| ☒ | ☐ | A full description of the statistical parameters including central tendency (e.g. means) or other basic estimates (e.g. regression coefficient) AND variation (e.g. standard deviation) or associated estimates of uncertainty (e.g. confidence intervals) |
| ☒ | ☐ | For null hypothesis testing, the test statistic (e.g. *F*, *t*, *r*) with confidence intervals, effect sizes, degrees of freedom and *P* value noted<br>*Give P values as exact values whenever suitable.* |
| ☒ | ☐ | For Bayesian analysis, information on the choice of priors and Markov chain Monte Carlo settings |
| ☒ | ☐ | For hierarchical and complex designs, identification of the appropriate level for tests and full reporting of outcomes |
| ☒ | ☐ | Estimates of effect sizes (e.g. Cohen's *d*, Pearson's *r*), indicating how they were calculated |

*Our web collection on statistics for biologists contains articles on many of the points above.*

## Software and code

Policy information about availability of computer code

| | |
|---|---|
| Data collection | no software was used |
| Data analysis | All analyses and visualizations were performed using Excel version 2307 and R Studio version R4.3.1 (R program for Statistical Computing). To summarize all actionable variants for the individual patients, an oncoprint was generated in R Studio using the Complex Heatmap package. The lollipop plot for BRCA1 and BRCA2 variants was adapted from cBioportal (genome build GRCh38) |

For manuscripts utilizing custom algorithms or software that are central to the research but not yet described in published literature, software must be made available to editors and reviewers. We strongly encourage code deposition in a community repository (e.g. GitHub). See the Nature Portfolio guidelines for submitting code & software for further information.

## Data

Policy information about availability of data

All manuscripts must include a data availability statement. This statement should provide the following information, where applicable:

- Accession codes, unique identifiers, or web links for publicly available datasets
- A description of any restrictions on data availability
- For clinical datasets or third party data, please ensure that the statement adheres to our policy

All data analyzed during this study are included in this published article and its supplementary information files. Availability of raw sequencing data is prohibited due to ethical consent restrictions.

## Research involving human participants, their data, or biological material

Policy information about studies with human participants or human data. See also policy information about sex, gender (identity/presentation), and sexual orientation and race, ethnicity and racism.

| | |
|---|---|
| Reporting on sex and gender | All patients in our cohort are of female sex, gender has not been recorded or specified. |
| Reporting on race, ethnicity, or other socially relevant groupings | We describe our patient cohort as mainly Caucasian race, representative of our population. However, individualized race was not recorded. |
| Population characteristics | Age and genetic alterations are the only individualized data reported. |
| Recruitment | This retrospective cohort study included 358 patients diagnosed with OC between January 2014 and December 2022 in 28 hospitals across East-Flandres and West-Flandres, for routine diagnostic tumor testing to provide PARP inhibitor therapy. |
| Ethics oversight | The study was approved by the ethics committee of Ghent University Hospital (ONZ-2023-0389) |

Note that full information on the approval of the study protocol must also be provided in the manuscript.

# Field-specific reporting

Please select the one below that is the best fit for your research. If you are not sure, read the appropriate sections before making your selection.

☒ Life sciences ☐ Behavioural & social sciences ☐ Ecological, evolutionary & environmental sciences

For a reference copy of the document with all sections, see nature.com/documents/nr-reporting-summary-flat.pdf

# Life sciences study design

All studies must disclose on these points even when the disclosure is negative.

| | |
|---|---|
| Sample size | This retrospective cohort study included 358 patients diagnosed with OC between January 2014 and December 2022. No sample size calculations were needed, we included all patients who underwent molecular testing. |
| Data exclusions | seven borderline or non-epithelial tumors were excluded for further analysis |
| Replication | this is an observational study, no intervention or experiments were needed |
| Randomization | this is an observational study, no intervention or experiments were needed |
| Blinding | this is an observational study, no intervention or experiments were needed |

# Reporting for specific materials, systems and methods

We require information from authors about some types of materials, experimental systems and methods used in many studies. Here, indicate whether each material, system or method listed is relevant to your study. If you are not sure if a list item applies to your research, read the appropriate section before selecting a response.

### Materials & experimental systems

| n/a | Involved in the study | |
|---|---|---|
| ☒ | ☐ | Antibodies |
| ☒ | ☐ | Eukaryotic cell lines |
| ☒ | ☐ | Palaeontology and archaeology |
| ☒ | ☐ | Animals and other organisms |
| ☒ | ☐ | Clinical data |
| ☒ | ☐ | Dual use research of concern |
| ☒ | ☐ | Plants |

### Methods

| n/a | Involved in the study | |
|---|---|---|
| ☒ | ☐ | ChIP-seq |
| ☒ | ☐ | Flow cytometry |
| ☒ | ☐ | MRI-based neuroimaging |

## Plants

| | |
|---|---|
| Seed stocks | no plants were used in this manuscript |
| Novel plant genotypes | no plants were used in this manuscript |
| Authentication | no plants were used in this manuscript |
